# Phosphorylated Platelet-Derived Growth Factor Receptor-Positive Cells With Anti-apoptotic Properties Accumulate in the Synovium of Patients With Rheumatoid Arthritis

**DOI:** 10.3389/fimmu.2019.00241

**Published:** 2019-02-15

**Authors:** Takashi Matsumura, Yuki Saito, Tomoyuki Suzuki, Atsushi Teramoto, Yasuhiro Ozasa, Toshihiko Yamashita, Mineko Fujimiya, Takako Saito-Chikenji

**Affiliations:** ^1^Department of Orthopaedic Surgery, Sapporo Medical University School of Medicine, Sapporo, Japan; ^2^Department of Anatomy, Sapporo Medical University School of Medicine, Sapporo, Japan

**Keywords:** rheumatoid arthritis, chronic inflammation, fibroblast-like synoviocyte, cadherin-11, platelet-derived growth factor receptor, apoptosis

## Abstract

Rheumatoid arthritis (RA) is an autoimmune disease caused by inflammation of the synovium and characterized by chronic polyarthritis that destroys bone and cartilage. Fibroblast-like synoviocytes (FLSs) in the synovium of patients with RA can promote cartilage and bone destruction by producing proteins such as matrix metalloproteinases and receptor activator of NF-κB ligand, thereby representing an important therapeutic target for RA. FLSs have several phenotypes depending on which cell surface proteins and adhesion factors are expressed. Identifying the cellular functions associated with different phenotypes and methods of controlling them are considered essential for developing therapeutic strategies for RA. In this study, synovial tissue was collected from patients with RA and control subjects who required surgery due to ligament injury or fracture. Immunohistological analysis was used to investigate the rates of positivity for phosphorylated platelet-derived growth factor receptor-αβ (pPDGFRαβ) and cadherin-11 (CDH11) expression, and apoptosis-related markers were assessed for each cell phenotype. Next, FLSs were isolated *in vitro* and stimulated with tumor necrosis factor-α (TNF-α) in addition to a combination of PDGF and transforming growth factor (2GF) to investigate pPDGFRαβ and CDH11 expression and the effects of the inhibition of TNF and cyclin-dependent kinase (CDK) 4/6 on FLSs. Immunohistological analysis showed a large percentage of pPDGFRαβ+CDH11– cells in the sub-lining layer (SL) of patients with RA. These cells exhibited increased B-cell lymphoma-2 expression, reduced TNF receptor-1 expression, resistance to cell death, and abnormal proliferation, suggesting a tendency to accumulate in the synovium. Further, *in vitro* 2GF stimulation of FLSs lowered, whereas 2GF + TNF stimulation increased the pPDGFRαβ/CDH11 ratio. Hypothesizing that FLSs stimulated with 2GF + TNF would accumulate *in vivo* in RA, we determined the therapeutic effects of TNF and CDK4/6 inhibitors. The TNF inhibitor lowered the pPDGFRαβ/CDH11 ratio, whereas the CDK4/6 inhibitor suppressed cell proliferation. However, a synergistic effect was not observed by combining both the drugs. We observed an increase in pPDGFRαβ+CDH11– cells in the SL of the RA synovium and accumulation of these cells in the synovium. We found that the TNF inhibitor suppressed FLS activity and the CDK4/6 inhibitor reduced cell proliferation.

## Introduction

Rheumatoid arthritis (RA) is an idiopathic form of arthritis that is both chronic and progressive and undergoes repeated stages of remission and flare-up (1). Advances in pharmacotherapies, such as with immunosuppressants and biological drugs, have raised remission rates, although inducing remission remains difficult in 30–40% of patients with RA, and joint destruction can progress even in remission (2). Moreover, flare-ups tend to occur when pharmacotherapy is halted, rendering it difficult to suspend medication. A fundamental therapy for RA is still lacking (3). In previous studies on RA patients and RA model animals, the accumulation of fibroblast-like synoviocytes (FLSs) in the joint synovium was reported as the primary cause of joint destruction (4, 5). Cells in the synovium are categorized morphologically as macrophage-like cells with non-fixed phagocytic capacity and FLSs, which have spindle shapes and form regular networks (6). There are several FLS phenotypes, including the expression of cell-adhesion factors cadherin-11 (CDH11) and gp38, as well as CD90, CD248, and others proteins that are also expressed as surface antigens on regular fibroblasts (7, 8). Among these, CDH11 is expressed in the lining layer (LL) and sub-lining layer (SL) of the synovium (9). FLSs in the LL produce a particularly large amount of matrix metalloproteinases (MMPs), which are known as the main agents of joint destruction, and are thus important therapeutic targets (4, 7, 10, 11). At present, one of the most effective drugs are tumor necrosis factor (TNF) inhibitors, the therapeutic mechanism of which is reported to be suppressing inflammation by reducing FLS influx into the LL (9). Nonetheless, TNF inhibitor therapy has little effect on FLSs in the SL (9) and has even been reported to cause fibrosis of the SL (12). Therefore, improving SL-FLS accumulation is thought to be difficult with existing therapies (13). Moreover, compared to the LL, the SL has more blood vessels, lymphocytes, plasma cells, and macrophages and is considered as a center of synovial inflammation (10). Therefore, gaining a deeper understanding of SL-FLS phenotypes and establishing methods of controlling them could help to develop novel therapies for RA.

Tissue fibrosis accompanying intractable chronic inflammation is associated with increased platelet-derived growth factor (PDGF) signaling and the proliferation and accumulation of PDGF receptor (PDGFR)-positive mesenchymal cells or fibroblasts in various organs, including the kidneys, liver, myocardium/skeletal muscle, and bone marrow (14–16). The synovium of RA patients has been reported to exhibit greater expression of phosphorylated PDGFRαβ (pPDGFRαβ), an activated form of PDGFR, than the synovium of osteoarthritis patients (17). PDGF signaling promotes cell survival and cell proliferation via the PI3K–At pathway and Ras–MAPK pathway (14), which strongly suggests that FLSs with a treatment-resistant, aggressive phenotype that accumulate in RA are pPDGFRαβ-positive. Therefore, this study aimed to investigate pPDGFRαβ expression in FLSs accumulated in the synovium during RA and to clarify their locations, phenotypes, and signals related to cell survival and cell death.

In this study, pPDGFRαβ-positive cells that proliferate specifically in the SL of RA exhibited low expression of TNF-receptor-1 (TNF-R1) and the cell-cycle suppressor p16, as well as high expression of B-cell lymphoma 2 (Bcl-2), which confers resistance to cell death. We also investigated resistance to cell death after increased pPDGFRαβ expression in FLSs through *in vitro* stimulation with PDGF-BB, TGF-β, and TNF-α, as well as candidate drugs for pPDGFRαβ-positive cells. We propose that a new therapeutic strategy can potentially be developed for RA by targeting pPDGFRαβ+CDH11– cells.

## Materials and Methods

### Patients and Tissue Samples

Experiments using human samples were approved by the institutional review board at the Sapporo Medical University (approval no., 292-3303), and all experiments were performed in accordance with relevant guidelines and regulations. Synovial tissues were obtained from patients undergoing arthroscopic or arthroplastic surgery at the Sapporo Medical University or Sapporo Maruyama Orthopedics Hospital, after informed consent was obtained from the patients. All subjects provided written informed consent in accordance with the Declaration of Helsinki. Twenty-five patients with RA fulfilling the American College of Rheumatology (ACR; formerly, the American Rheumatism Association) criteria were included in this study. In addition, 13 patients who required arthroscopic surgery for ligament injury or fracture were included as control patients with acute inflammation. Acute inflammation was defined as that occurring less than 8 weeks after injury, as in previous studies (18). The clinical features of the patients who donated samples are summarized in Table 1.

**Table 1:** Characteristics of patients with RA and acute inflammation (control subjects).

	**RA**	**Control**
	***N* = 25**	***N* = 13**
Age (years)	58.2 (25–83)	36.6 (20–61)
Gender (% female)	89%	31%
Disease duration	11.7 (1–40) years	19.8 (3–56) days
Treatment	Prednisolone 15 (63%)	–
	Methotrexate 8 (33%)	–
	Biological drugs 8 (33%)	–
ACPAs, positive patients (%)	11/14 (79%)	–
RF, positive patients (%)	18/23 (82%)	–
CRP (mg/dL)	1.3 (0.1–5.8)	–
MMP3 (ng/mL)	206.2 (53–596)	–

*ACPAs, anti-citrullinated protein antibodies; control, trauma patients; CRP, C-reactive protein; MMP3, matrix metalloproteinase 3; RA, patients with rheumatoid arthritis; RF, rheumatoid factor*.

### Immunohistochemistry of Tissues

For immunohistochemistry studies, tissues were fixed in 4% paraformaldehyde overnight. The following day, the tissues were transferred to 20% sucrose in phosphate buffer and incubated overnight, frozen in OCT compound by using liquid nitrogen, and stored at −80°C until use. Cryosections (8-μm thick) were prepared using a cryostat. The sections were stained with hematoxylin and eosin (HE). For immunohistochemistry, the sections were incubated in 0.01 M phosphate-buffered saline (PBS) containing 0.3% Triton-X (PBS-T) and 2% bovine serum albumin (BSA) for 60 min at room temperature (RT). After the sections were washed with 0.01 M PBS-T, they were incubated with primary antibodies at 4°C overnight, followed by staining with secondary antibodies. Staining was performed using primary antibodies against anti-pPDGFRα(Tyr849)/β(Tyr857; 1:75; Cell Signaling Technology, Danvers, MN, USA), anti-cadherin-11 (15 μg/mL; R&D Systems, Minneapolis, MN, USA), TNF-R1 (1:500; Thermo Fisher Scientific, San Diego, CA, USA), Bcl-2 (1:50; BD Biosciences, Franklin Lakes, NJ, USA), p16 (1:200, Abcam, Cambridge, UK), and p53 (1:50; Santa Cruz Biotechnology, Santa Cruz, CA, USA), CD45 (1:1000; Abcam). For secondary antibodies, we used Alexa Fluor 488-conjugated IgG (1:100; Jackson ImmunoResearch, West Grove, PA, USA), Alexa Fluor 647-conjugated IgG (1:100; Jackson ImmunoResearch), and Cy3-conjugated IgG (1:100; Merck Millipore, Darmstadt, Germany). Nuclei were stained using 4′6-dimidino-2-phenylindole (DAPI; 1:1000; Dojindo, Kumamoto, Japan). Sections were observed using a confocal laser scanning microscope (Nikon/A1; Nikon, Tokyo, Japan) and fluorescence microscopy (BZ-X800, Keyence Corp., Osaka, Japan). NIS elements (Nikon) and BZ-X800 analysis application (Keyence Corp.) were used for cell analysis. Images were acquired from eight different regions of each tissue section. Settings for one staining experiment were maintained across all experiments. LL and SL were distinguished by morphology for each region by using low-magnification imaging. The cell depth of the LL was assessed for 3 regions of each high-power field of the synovium, and the LL thickness was expressed as the mean number of cells in depth. High-magnification imaging was used to confirm the identity and analyze the distribution of cells. DAPI staining was used to count separately pPDGFRαβ-positive and CDH11-positive cells and classify cells into three groups as follows: pPDGFRαβ+CDH11–, pPDGFRαβ-CDH11+, and pPDGFRαβ+CDH11+ cells; next, the relative abundance of each cell type was evaluated. Similarly, cells positive for TNF-R1, Bcl-2, p16, p53, or CD45 expression were counted. Briefly, we classified cells into pPDGFRαβ+CDH11–, pPDGFRαβ-CDH11+, and pPDGFRαβ+CDH11+ cells by overlay image of DAPI filter channel (excitation 360 nm, emission 460 nm; DAPI), GFP filter channel (excitation 470 nm, emission 525 nm; CDH11) and Cy5 filter channel (excitation 620 nm, emission 700 nm; pPDGFRαβ); next, the TRITC filter channel (excitation 545 nm, emission 605 nm; TNF-R1, Bcl-2, p16, p53 and CD45) image was overlayed on the overlay image of DAPI, GFP and Cy5 filter channel to evaluate cells positive for TNF-R1, Bcl-2, p16, p53 and CD45 in pPDGFRαβ+CDH11–, pPDGFRαβ-CDH11+, and pPDGFRαβ+CDH11+ cells. Two researchers independently assessed the number of cells positive for each marker. Intraclass correlation coefficients (ICCs) for pPDGFRαβ and CDH11 reflected good (ICC = 0.82) internal consistency.

### Cell Preparation and Cell Proliferation Assays

Synoviocytes were isolated from synovial tissue by using standard procedures (19). Synoviocytes from patients with RA were digested with Liberase TM Research Grade (Sigma–Aldrich) for 90 min at 37°C. The digested synoviocyte slurries were filtered through a 100 μm cell strainer (EASYstrainer™ Cell; Greiner Bio-One, Kremsmünster, Austria). Cells were suspended in the growth medium consisting of Dulbecco's modified Eagle's medium (Sigma–Aldrich) supplemented with 10% fetal bovine serum, 100 U/mL penicillin, and 100 μg/mL streptomycin and incubated at 37°C in 5% CO_2_. Cells at passages 3 to 6 were used in all experiments.

For cytokine stimulation, cells were treated with or without various concentrations (1, 10, and 100 ng/mL) of PDGF-BB (BioLegend; San Diego, CA, USA), TGF-β (BioLegend), or TNF-α (BioLegend) for 2 days, as per our approved experimental design. The role of therapeutic drugs was determined by treating cells stimulated with cytokines with 10, 25, or 50 μg/mL etanercept (Pfizer, NY, USA) or 7.5 μM or 15 μM palbociclib (Sigma–Aldrich), or a combination of 25 μg/mL etanercept and 7.5 μM palbociclib for 1 day, as per our experimental design. These doses of cytokines and inhibitors were selected based on the findings of previous studies (20–23). WST-8 assays were performed to assess cell proliferation by using the Cell Counting Kit-8 (CK04; Dojindo).

### In-cell Enzyme-Linked Immunosorbent Assays

Cells were cultured in 96-well plates and treated with various concentrations of PDGF-BB, TGF-β, and/or TNF-α in combination with palbociclib and/or etanercept for 2 days. Cultured cells were fixed with 4% paraformaldehyde for 15 min at RT and then incubated in 2% BSA in PBS-T for 60 min at RT. After the cells were washed with PBS-T, they were incubated for 2 h at RT with a primary Alexa Fluor 488-conjugated anti-PDGFRA(Tyr849)/PDGFRB(Tyr857) antibody (1:20; Bioss, Boston, MA, USA) and a primary APC-conjugated anti-cadherin-11 antibody (1:80; BioLegend). Nuclei were stained with DAPI (1:1000; Dojindo). The fluorescence intensities of pPDGFRαβ and CDH11 staining were measured using a microplate reader (INFINITE M1000 PRO; Tecan Trading AG, Switzerland) and normalized based on the DAPI staining intensity.

### Statistical Analysis

Normality was assessed using the Shapiro–Wilk test. An unpaired *t*-test was used to assess differences in the percentage of pPDGFRαβ- and CDH11-positive cells between the control and RA synovium. One-way analysis of variance was used to assess percentage differences in histological cell staining under each culture condition. The *P*-values for multiple comparisons were adjusted using the Tukey–Kramer test. Pearson's correlation coefficient was used to assess the correlation. Dunnett's test was used to assess the concentration-dependent effect of each inhibitor. Statistical analyses were performed using EZR, a graphical user interface for R (The R Foundation for Statistical Computing, Vienna, Austria) (24). Two-sided *P*-values less than 0.05 were considered statistically significant.

## Results

### Accumulation of pPDGFRαβ+CDH11– Cells in the SL of the Synovium of Patients With RA

**Figure 1:**
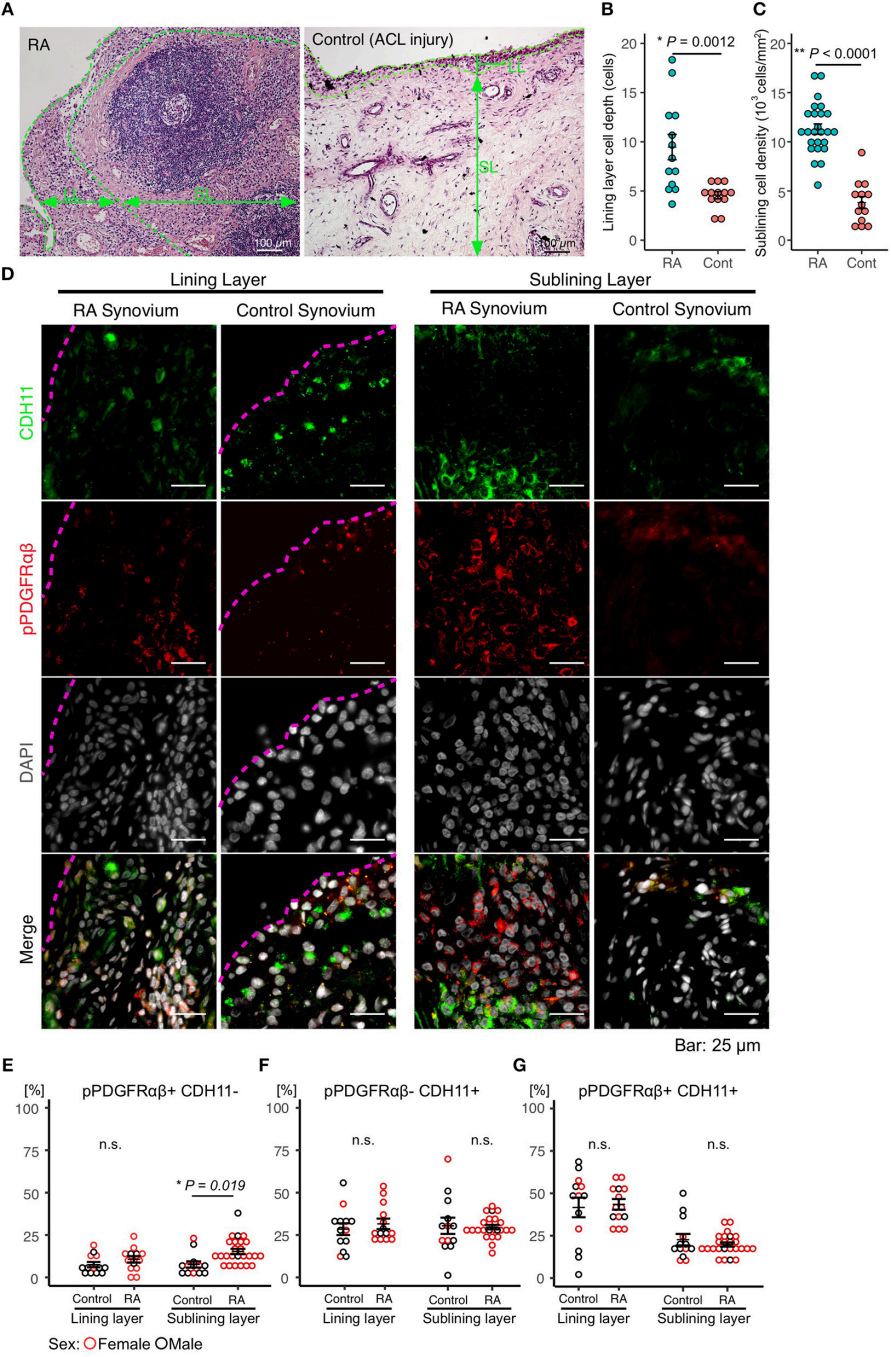
Accumulation of pPDGFRαβ+CDH11– cells in the sub-lining layer (SL) of the synovium in rheumatoid arthritis (RA). **(A)** HE staining of the synovial tissue collected from patients with RA and control subjects. Thickening of the lining layer (LL) and an elevated cell count in the SL were observed in RA. **(B)** the RA group's LL exhibited an increased cell depth, **(C)** and the cell count was elevated in the SL. **(D)** representative images of pPDGFRαβ and CDH11 staining of synovial tissue from controls and patients with RA, and quantitative data on 3 cell populations (pPDGFRαβ+CDH11–, pPDGFRαβ-CDH11+, and pPDGFRαβ+CDH11+ cells). The dotted line shows the margin of LL. **(E–G)** the percentages of pPDGFRαβ+CDH11–, pPDGFRαβ-CDH11+, and pPDGFRαβ+CDH11+ cells in the LL did not differ between the RA and control groups. **(E–G)** the percentage of pPDGFRαβ+CDH11– cells in the SL was significantly higher in the RA group than in the control group (*P* = 0.019). The quantitative data show the percentages of pPDGFRαβ+CDH11–, pPDGFRαβ-CDH11+, and pPDGFRαβ+CDH11+ cells out of the total number of cells. An unpaired *t*-test was used for statistical analysis. The significance level was *P* < 0.05.

Immunostaining was performed to identify FLSs in the synovium samples obtained during surgery from patients with RA (*n* = 25) and those having acute inflammation (*n* = 13). The mean age of the patients with RA was 58.2 (25–83) years, and their mean disease duration was 11.7 (1–40) years. The demographic data, including drug history, of the patients are shown in Table 1. HE staining was performed to compare the synovial tissues between the RA and control groups. Compared to that in the control group, increased LL thickening and excess cells in the SL were observed in RA (Figures 1A–C, Supplemental Figure 1). Differences between the RA and control groups regarding FLS protein expression and location were determined by performing immunostaining against CDH11 (5), a typical FLS marker, and pPDGFRαβ, which increases specifically in the synovium of RA and is positive in RA-FLS (17). Compared to control subjects, patients with RA exhibited LL thickening and a clearly elevated cell depth (Figures 1A,B), but no differences were noted in the percentages of pPDGFRαβ+CDH11–, pPDGFRαβ-CDH11+, or pPDGFRαβ+CDH11+ cells (Figures 1D–G). In the SL, RA exhibited a clearly elevated cell count (*P* < 0.0001; Figure 1C) and an increased percentage of pPDGFRαβ+CDH11– cells (*P* = 0.019; Figures 1D,E), but no differences were noted in the percentages of pPDGFRαβ-CDH11+ or pPDGFRαβ+CDH11+ cells (Figures 1D,F,G). Whether the pPDGFRαβ+ cells are of hematopoietic lineage was determined by performing immunostaining against CD45, pPDGFRαβ, and CDH11 in the SL; the percentage of CD45-positive cells per total cells was 33.9 ± 4.9% and that of cells double positive for CD45 and pPDGFRαβ was 1.28 ± 1.2% (Supplemental Figure 2).

### Characteristics of pPDGFRαβ+CDH11– Cells Accumulating in the Synovium of Patients With RA

**Figure 2:**
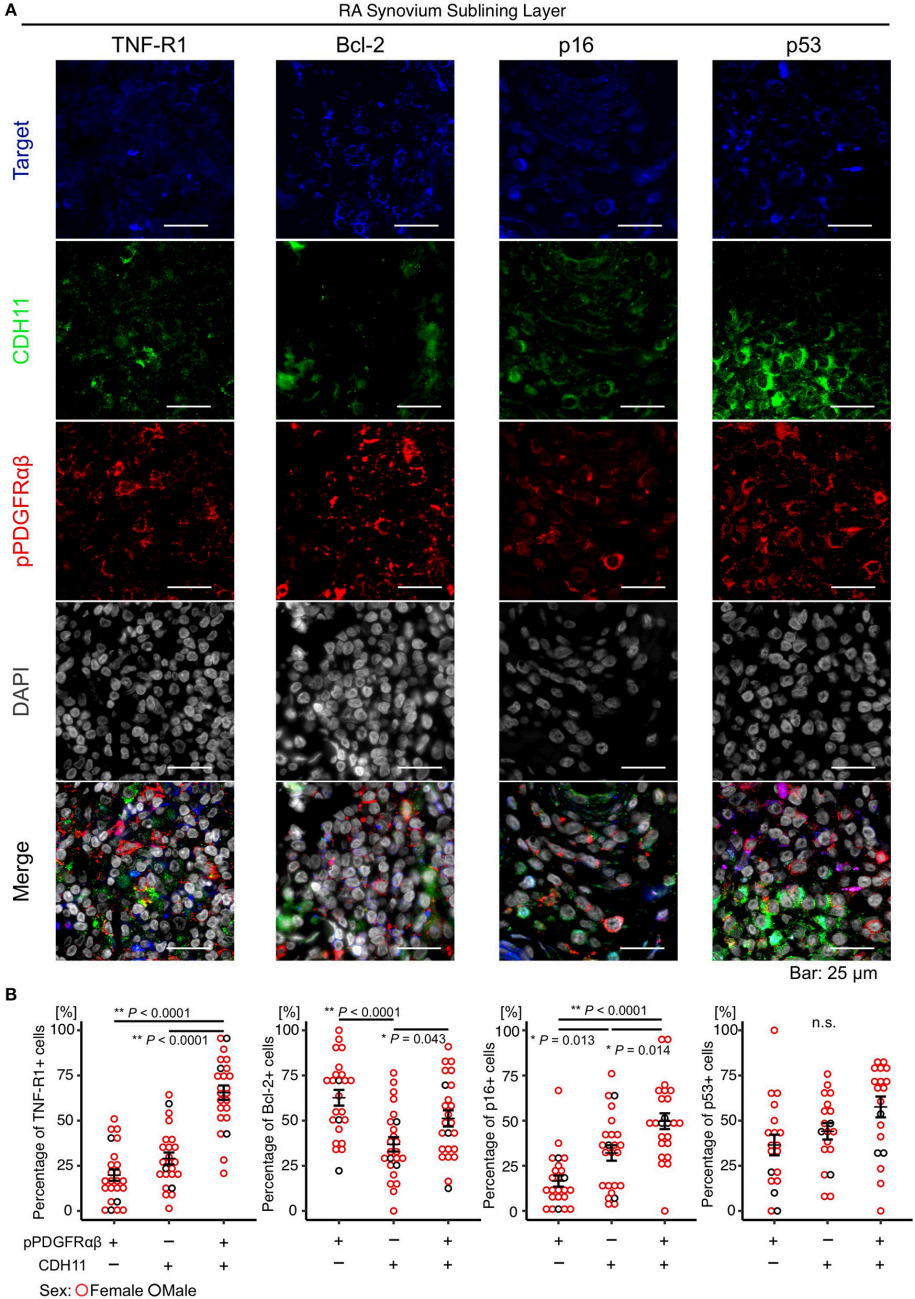
The pPDGFRαβ+CDH11– cells that accumulated in the SL of the synovium in RA exhibited reduced TNF-R1 and p16 expression and increased Bcl-2 expression. **(A)** representative staining images showing pPDGFRαβ, CDH11, TNF-R1, Bcl-2, p16, and p53 expression in the RA-SL. **(B)** quantitative expression data for TNF-R1, Bcl-2, p16, and p53 in 3 cell populations (pPDGFRαβ+CDH11–, pPDGFRαβ-CDH11+, pPDGFRαβ+CDH11+ cells). The percentage of TNF-R1-positive cells among pPDGFRαβ+CDH11– cells was significantly lower than that among pPDGFRαβ-CDH11+ and pPDGFRαβ+CDH11+ cells. The percentage of Bcl-2-positive cells among pPDGFRαβ+CDH11– cells was significantly higher than that among pPDGFRαβ-CDH11+ cells. The percentage of Bcl-2-positive cells among pPDGFRαβ+CDH11+ cells was significantly higher than that among pPDGFRαβ-CDH11+ cells. The percentage of p16-positive cells among pPDGFRαβ+CDH11– cells was significantly lower than that among pPDGFRαβ-CDH11+ and pPDGFRαβ+CDH11+ cells. p53 expression did not differ significantly between the 3 cell populations. The quantitative data show the TNF-R1-, Bcl-2-, p16-, and p53-positive rates for the pPDGFRαβ+CDH11–, pPDGFRαβ-CDH11+, and pPDGFRαβ+CDH11+ cells. One-way analysis of variance (ANOVA) was used for statistical analysis, and Tukey–Kramer method was used for intergroup comparisons. The significance level was *P* < 0.05.

The attributes of pPDGFRαβ+CDH11– cells accumulating in the RA-SL were clarified by comparing the expression levels of TNF-R1, Bcl-2, p16, and p53 between pPDGFRαβ+CDH11–, pPDGFRαβ-CDH11+, and pPDGFRαβ+CDH11+ cells in the RA-SL (Figure 2A). The TNF-R1, Bcl-2, p16, and p53 proteins regulate cell death and cell proliferation (25, 26). Increased expression of Bcl-2 and decreased expression of TNF-R1, p16, and p53 are reported to induce tissue fibrosis through apoptosis resistance and hyperproliferation (25, 26). The percentage of TNF-R1-positive cells among pPDGFRαβ+CDH11– cells was significantly lower than that among pPDGFRαβ+CDH11+ cells (*P* < 0.0001; Figure 2B). The percentage of Bcl-2-positive cells was significantly higher among pPDGFRαβ+CDH11– cells than among pPDGFRαβ-CDH11+ cells (*P* < 0.0001; Figure 2B); in contrast, the percentage in pPDGFRαβ-CDH11+ cells was significantly lower than that in pPDGFRαβ+CDH11+ cells (*P* < 0.043; Figure 2B). The percentage of p16-positive cells among pPDGFRαβ+CDH11– cells was significantly lower than that among pPDGFRαβ+CDH11– cells and pPDGFRαβ+CDH11+ cells (*P* = 0.013 and *P* < 0.0001, respectively; Figure 2B). Significant differences were not observed in the percentage of p53-positive cells. The above findings show that pPDGFRαβ+CDH11– cells exhibited low TNF-R1 and p16 expression and high Bcl-2 expression. Further, the apoptotic and hyperproliferative features of the three types of cells were better understood by performing immunohistochemistry analysis in the RA and control synovium. We found that Bcl-2 expression in RA was significantly higher than that in the control in pPDGFRαβ+CDH11–, pPDGFRαβ-CDH11+, and pPDGFRαβ+CDH11+ populations (*P* = 0.0037, *P* < 00001, and *P* = 0.0012, respectively; Supplemental Figure 3). In the RA group, the three types of cells showed a significant difference; however, in the control group, Bcl-2 expression was not markedly different among the three types of cells. This suggests that pPDGFRαβ+ cells have more anti-apoptotic property compared to CDH11-positive cells in RA. In the control, p16 expression of the pPDGFRαβ+CDH11+ population was significantly higher than that of the other cell types, and p16 expression in the pPDGFRαβ-CDH11+ population was higher than that in the pPDGFRαβ+CDH11– population (pPDGFRαβ+CDH11+ > pPDGFRαβ-CDH11+ > pPDGFRαβ+CDH11–; *P* < 0.0001 and *P* < 0.0001, respectively; Supplemental Figure 3); this tendency was the same as that in the RA group (Figure 2B). However, p16 expression in the pPDGFRαβ+CDH11+ population in RA was significantly lower than that of the control (*P* = 0.039; Supplemental Figure 3), suggesting that the pPDGFRαβ+CDH11+ population of RA might have greater proliferation capacity than in control.

### Correlations Between the Therapeutic Duration and Expression Rates of Various Markers in Patients With RA

**Figure 3:**
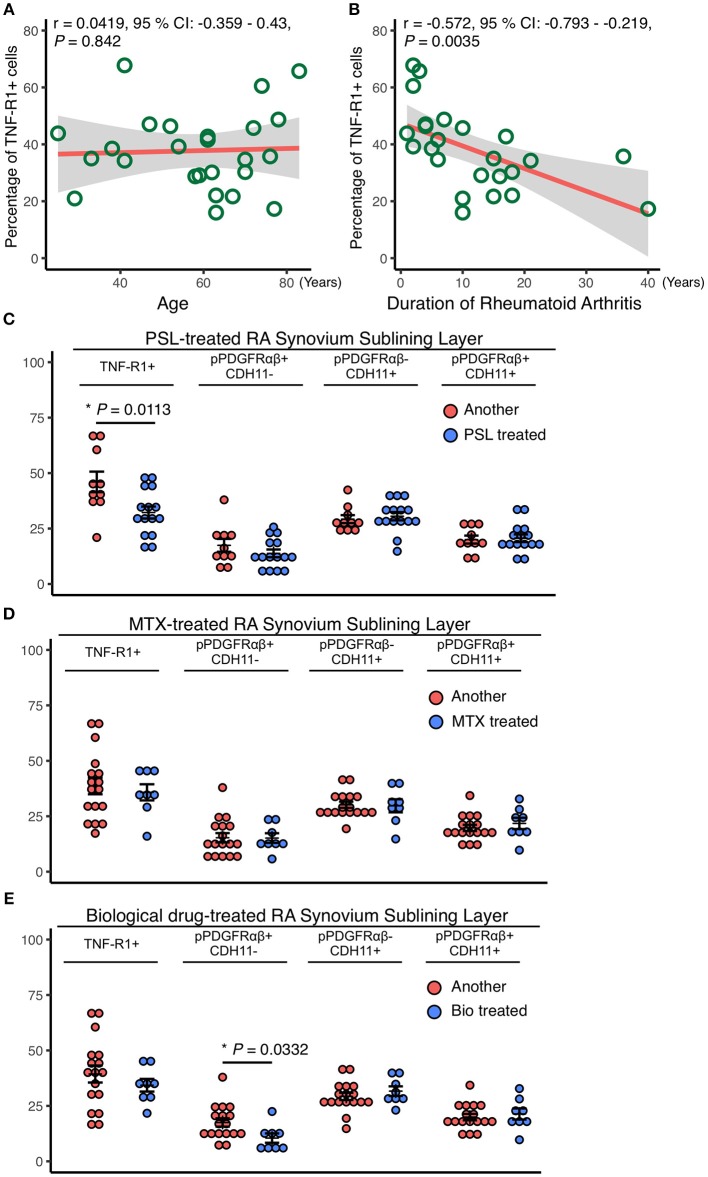
Reduced TNF-R1 expression in the RA-SL was associated with a longer RA duration and the use of prednisolone (PSL). Correlations between the rate of TNF-R1 positivity in the SL and **(A)** the age of RA patients and **(B)** RA duration. Although the TNF-R1 positivity rate did not correlate with age, a negative correlation with RA duration was observed. **(C–E)** the TNF-R1-positive rate in the SL was compared with the percentages of pPDGFRαβ+CDH11–, pPDGFRαβ-CDH11+, and pPDGFRαβ+CDH11+ cells, depending on whether methotrexate (MTX) or biological drugs were used. **(C)** although the use of PSL reduced TNF-R1 expression in the SL, significant differences were not observed in the percentages of pPDGFRαβ+CDH11–, pPDGFRαβ-CDH11+, or pPDGFRαβ+CDH11+ cells. **(D)** MTX use was not associated with significant differences in the TNF-R1 expression rate or in the percentages of any of the cell populations. **(E)** the TNF-R1 expression rate did not differ significantly with the use of biological drugs, although a significant decrease in pPDGFRαβ+CDH11– cells was observed. Correlations were examined statistically by using Pearson's correlation coefficient, and unpaired *t*-test was used to compare the effects of drugs. The significance level was *P* < 0.05.

The relationships between RA patient characteristics and RA-SL attributes were determined by investigating the correlations of TNF-R1, pPDGFRαβ, and CDH11 expression with the treatment duration and therapeutic agents. TNF-R1 expression was not correlated with age (*r* = 0.042, *P* = 0.842; Figure 3A), although it was negatively correlated with RA treatment duration (*r* = −0.572, *P* = 0.0035; Figure 3B). The longer the duration of RA treatment with prednisolone (PSL), methotrexate (MTX), or biological drugs, the lower was the expression of TNF-R1 in SL cells. TNF-R1 expression was significantly lower following PSL therapy than after MTX or biological drug therapy (*P* = 0.0113; Figure 3C). However, TNF-R1 expression was not significantly different between MTX and biological drug therapy (Figures 3D,E). The percentage of pPDGFRαβ+CDH11–, pPDGFRαβ-CDH11+, and pPDGFRαβ+CDH11+ cells were not significantly different after PSL or MTX therapy (Figures 3D,E), although biological drug therapy was associated with a significantly lower percentage of pPDGFRαβ+CDH11– cells (*P* = 0.0332; Figure 3E).

### Increased pPDGFRαβ Expression in RA-FLSs After PDGF-BB, TGF-β, and TNF-α Stimulation

**Figure 4:**
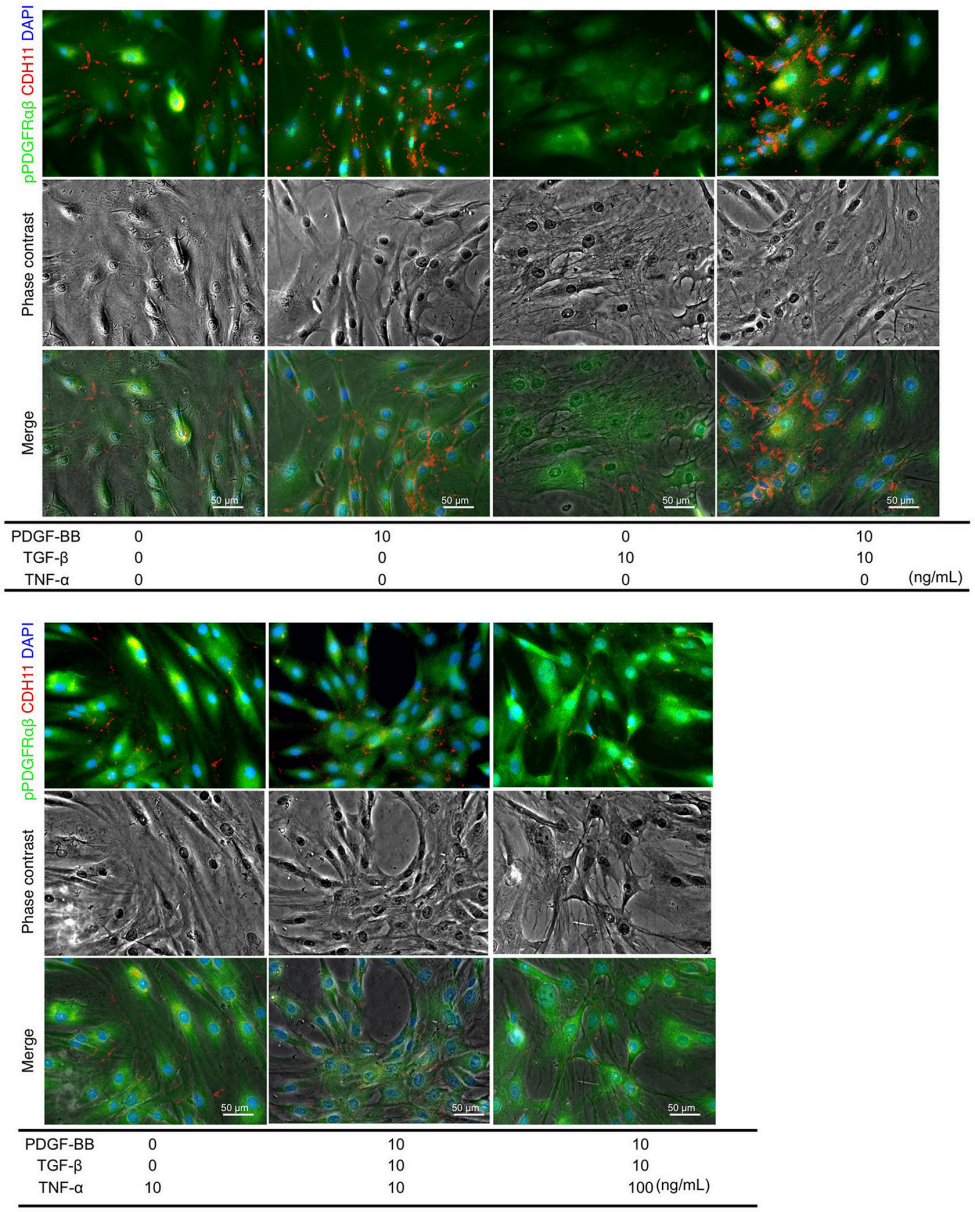
pPDGFRαβ and CDH11 expression by PDGF-BB, TGF-β, and TNF-α stimulation of RA-FLS. The effects of PDGF-BB, TGF-β stimulation alone, PDGF-BB and TGF-β (2GF) stimulation, 2GF + 10 ng/mL TNF, and 2GF + 100 ng/mL TNF stimulation on RA-FLSs were investigated. Representative images of immunostaining for pPDGFRαβ (green) and CDH11 (red) expression with FLSs stimulated with PDGF, TGF-β, and TNF in each combination.

**Figure 5:**
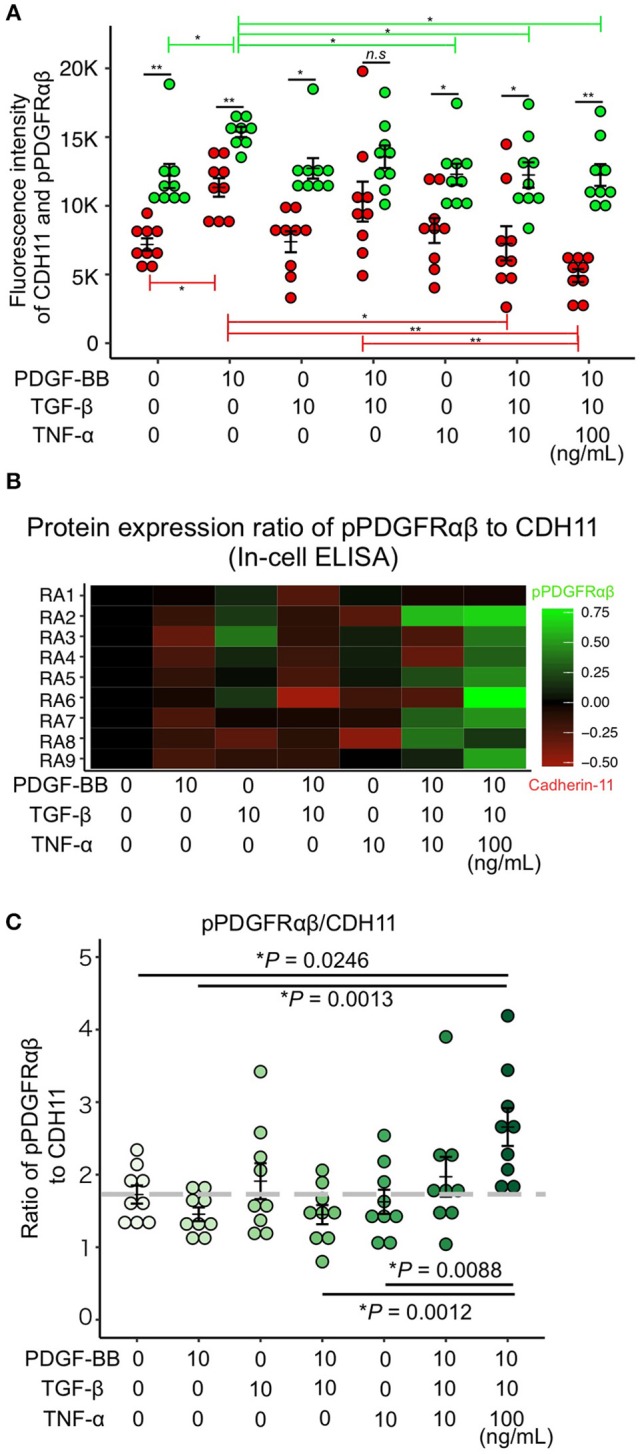
PDGF-BB, TGF-β, and TNF-α stimulation of RA-FLS resulted in predominant pPDGFRαβ expression. **(A–C)** quantitative data for pPDGFRαβ and CDH11 expression obtained by performing in-cell ELISAs. Stimulation with TNF in addition to 2GF reduced CDH11 expression, but not pPDGFRαβ expression. Stimulation with 2GF reduced the pPDGFRαβ/CDH11 ratio and elevated CDH11 expression. Stimulation with 2GF + TNF caused concentration-dependent increases in the pPDGFRαβ/CDH11 ratio and elevated pPDGFRαβ expression. One-way ANOVA was used for statistical analysis, and the Tukey–Kramer method was used to compare the groups. The significance level was *P* < 0.05. ^*^P < 0.05; ^**^P < 0.01.

PDGF-BB and TGF-β (2GF) stimulation with or without TNF-α were performed to reproduce the pPDGFRαβ-predominant environmental characteristic of RA-FLSs *in vitro*. PDGF-BB, TGF-β, and TNF-α are typical factors that exacerbate the pathology of RA. Because 2GF + TNF stimulation conferred aggressive phenotypes to FLSs with elevated expression of IL-6, IL-8, and other inflammatory cytokines and factors such as MMP3 (20, 21), we hypothesized that 2GF + TNF-α would increase the expression level of pPDGFRαβ in FLSs. The number of live cells did not differ between 2GF and 2GF + TNF (10 ng/mL or 100 ng/mL) stimulation of FLSs (*P* = 0.0631; Supplemental Figure 4). Immunostaining against pPDGFRαβ and CDH11 was performed to examine pPDGFRαβ and CDH11 expression after 2GF and 2GF + TNF stimulation (Figure 4). In-cell enzyme-linked immunosorbent assays were also performed to quantify CDH11 and pPDGFRαβ expression. Stimulation with PDGF-BB significantly increased CDH11 and pPDGFRαβ expression compared to that without stimulation and 2GF + TNF stimulation (Figures 4, 5A). Stimulation with 2GF + TNF (100 mg/mL) significantly decreased CDH11 expression, but did not significantly affect pPDGFRαβ expression (*P* = 0.002 and *P* = 0.9123, respectively; Figures 4, 5A). Further, stimulation with 2GF + TNF increased the pPDGFRαβ/CDH11 ratio in a TNF-concentration-dependent manner versus 2GF stimulation, making pPDGFRαβ expression predominant (Figures 5B,C; *P* < 0.0001). Because 2GF stimulation caused predominant CDH11 expression, 2GF stimulation appeared to generate a phenotype similar to FLSs in a state of acute inflammation. This is because, during acute inflammation, the synovium exhibits increased CDH11-positive cells, but few cells expressing pPDGFRαβ (17). Further, because 2GF + TNF stimulation increased the pPDGFRαβ/CDH11 ratio, this was thought to represent a phenotype that resembled pPDGFRαβ+CDH11– cells that accumulate in the RA-SL *in vivo*.

### Effects of the TNF Inhibitor, Etanercept

**Figure 6:**
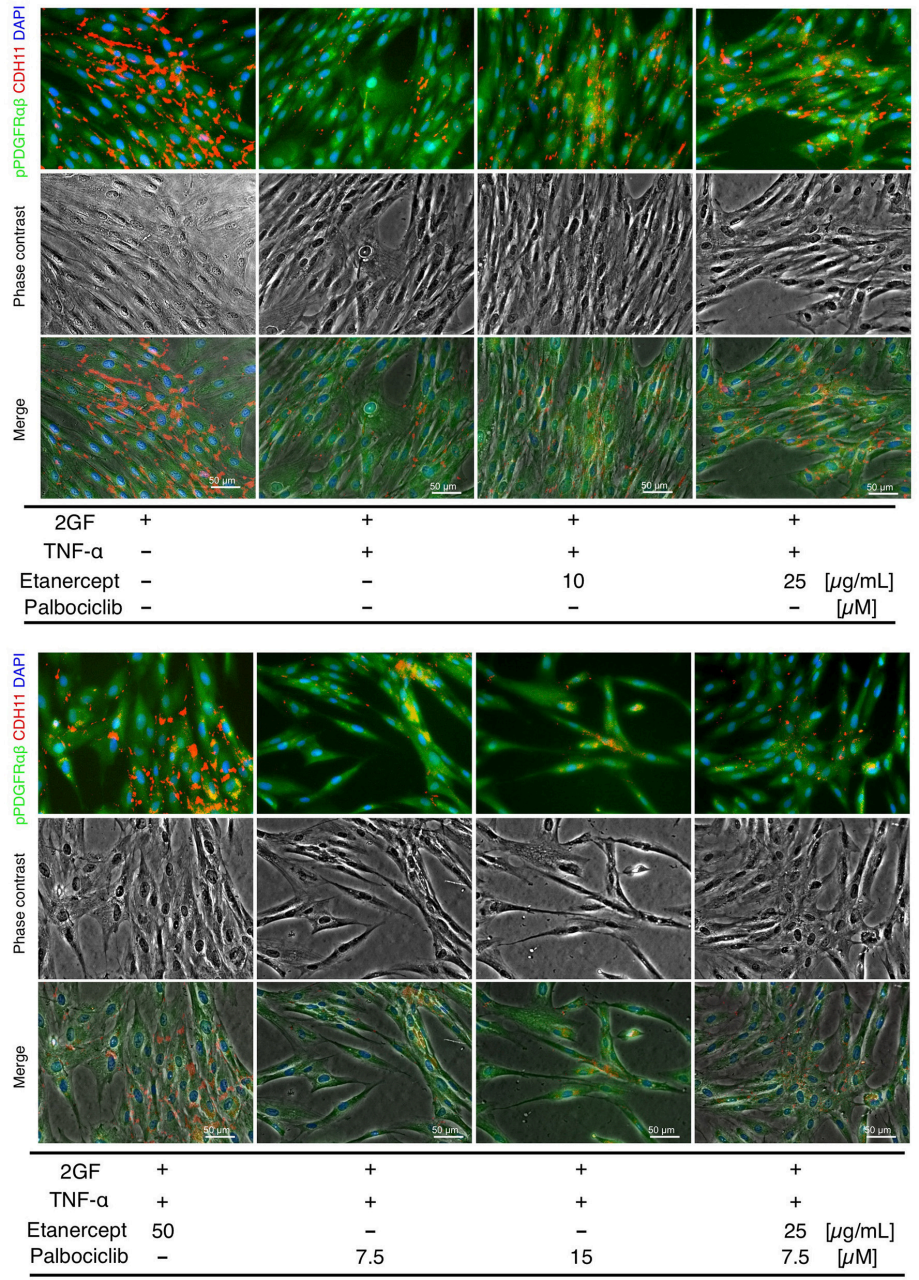
pPDGFRαβ and CDH11 expression by 2GF + TNF and a TNF inhibitor (etanercept) and a CDK4/6 inhibitor (palbociclib) in RA-FLSs. Representative images of immunostaining for pPDGFRαβ and CDH11 expression with FLSs stimulated with 2GF + TNF and the TNF and CDK4/6 inhibitors in each combination.

**Figure 7:**
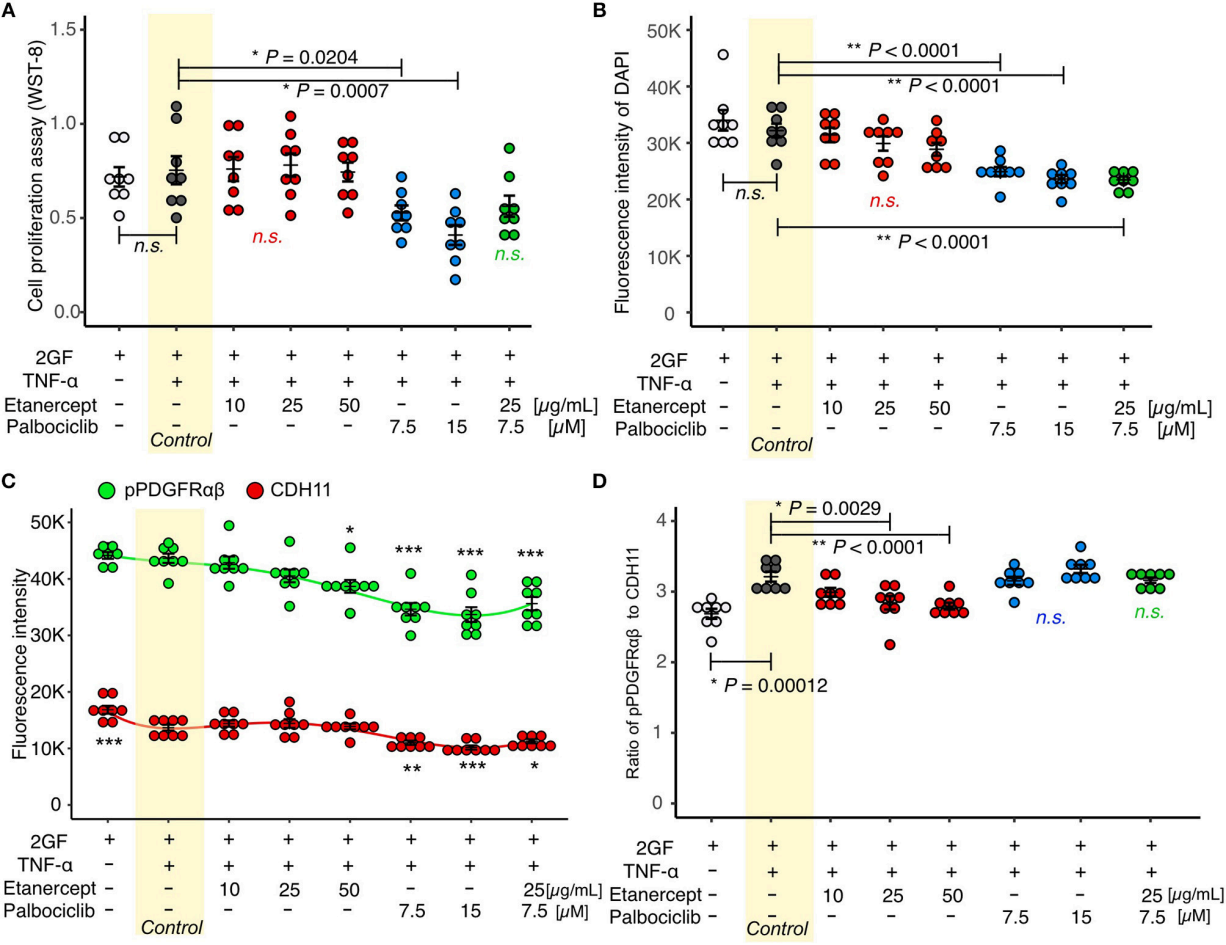
Etanercept suppressed pPDGFRαβ expression in RA-FLSs stimulated with 2GF + TNF, and palbociclib reduced the cell count. **(A)** cell proliferation assay and **(B–D)** quantitative cell count and pPDGFRαβ/CDH11 ratio data obtained by performing in-cell ELISAs with FLSs stimulated with 2GF + TNF and exposed to different concentrations of etanercept and palbociclib. Adding etanercept to FLSs stimulated with 2GF + TNF did not significantly decrease the number of live cells, but significantly reduced the pPDGFRαβ/CDH11 ratio. Adding palbociclib significantly reduced the number of live cells, but did not significantly change the pPDGFRαβ/CDH11 ratio. Etanercept + palbociclib significantly reduced the number of live cells, but did not significantly change the pPDGFRαβ/CDH11 ratio. Dunnett's test was used to statistically analyze differences occurring with 2GF + TNF stimulation. The significance level was *P* < 0.05. ^*^P < 0.05; ^**^P < 0.01; ^***^P < 0.001.

Concomitant TNF and 2GF stimulation caused predominant pPDGFRαβ expression in FLSs, suggesting that TNF-α inhibited CDH11 expression, but did not affect pPDGFRαβ expression in the FLSs. Next, we investigated whether a TNF inhibitor (etanercept), most commonly used in RA therapy, could restore the pPDGFRαβ/CDH11 ratio after 2GF + TNF stimulation (Figure 6). Adding etanercept to FLSs stimulated with 2GF + TNF did not lead to a significant decrease in the number of live cells, according to WST-8 assays (*P* = 0.979; Figure 7A). Further, adding etanercept did not reduce the total cell count (*P* = 0.247; Figure 7B). However, etanercept at a concentration of 50 μg/mL significantly reduced pPDGFRαβ expression (*P* = 0.0056; Figure 7C), and that at a concentration of 25 or 50 μg/mL significantly reduced the pPDGFRαβ/CDH11 ratio (*P* = 0.00294 and *P* < 0.001, respectively; Figure 7D). Since a TNF inhibitor could reduce the pPDGFRαβ expression and pPDGFRαβ/CDH11 ratio, this drug could suppress FLSs with pPDGFRαβ expression, which is thought to have an aggressive phenotype that contributes to RA.

### Effects of the CDK4/6 Inhibitor, Palbociclib

While the TNF inhibitor caused a reduction in the pPDGFRαβ/CDH11 ratio, it had no effect on the cell count. We confirmed an excess accumulation of cells in the SL of the synovium of patients with RA *in vivo* (Figure 1A). This finding indicates that, in addition to changing the FLS phenotype, reducing the cell count is essential for RA therapy. The cell senescent factor p16 exhibited reduced expression in the accumulated cells *in vivo* (Figures 2A,B). Because p16 negatively regulates CDK4/6 (25), we surmised that reduced p16 expression in RA synovial tissue results in elevated expression of CDK4/6. Reduced p16 expression and increased CDK4/6 expression are often observed in cancer cells and cancer-associated fibroblasts (CAFs) in breast cancer and head and neck cancer and can induce hyperproliferation and cancer cell spread (27, 28). Further, CDK4/6 inhibitors can effectively suppress cell proliferation and the epithelial-mesenchymal transition (EMT) (22). Therefore, we investigated whether the CDK4/6 inhibitor palbociclib would reduce the number of FLSs (Figure 6). When palbociclib was added at a concentration of 7.5 μM or 15 μM to FLSs stimulated with 2GF + TNF, significant reduction in the number of live cells (*P* = 0.0204 and *P* = 0.0007, respectively; Figure 7A) and reduction in the total cell count (*P* < 0.0001 and *P* < 0.0001, respectively; Figure 7B) were observed in WST-8 assays. Adding palbociclib significantly decreased the total expression level of pPDGFRαβ and CDH11, but did not significantly decrease the pPDGFRαβ/CDH11 ratio and the pPDGFRαβ and CDH11 expression level per cell (Figures 7C,D, Supplemental Figure 5). These findings showed that CDK4/6 inhibition could suppress FLS proliferation and reduce the cell count; therefore, palbociclib can be expected to exert a therapeutic effect on accumulated cells.

### Effects of Combination Treatment With a TNF Inhibitor and a CDK4/6 Inhibitor

To treat an increased number of FLSs that become pPDGFRαβ-predominant owing to 2GF + TNF stimulation, we investigated the combined effects of a TNF inhibitor (which reduced the expression of pPDGFRαβ) and a CDK4/6 inhibitor (which reduced the cell count; Figure 6). WST-8 assays showed that the cell count did not decrease significantly (*P* = 0.0783; Figure 7A), although a reduction in the total cell count was observed when in-cell enzyme-linked immunosorbent assays were performed (*P* < 0.001; Figure 7B). Moreover, the combination treatment with etanercept and palbociclib significantly decreased the total expression level of pPDGFRαβ and CDH11 (*P* < 0.0001; Figure 7C), but a significant difference in the pPDGFRαβ/CDH11 ratio (which was observed with a TNF inhibitor alone) was not found with combination treatment (*P* = 0.916; Figure 7D). The effects of combined treatment with a CDK4/6 inhibitor and a TNF inhibitor were similar to those of treatment with the CDK4/6 inhibitor alone, which indicates that including a TNF inhibitor had little effect.

## Discussion

FLSs play a role in the maintenance of homeostasis of the synovial fluid and a healthy synovium. In the case of acute inflammation such as due to injury, FLSs are activated; they proliferate and mobilize immune cells in the early stages of inflammation, exerting anti-inflammatory effects as inflammation subsides and participating in synovial remodeling by producing the extracellular matrix (ECM). FLSs are essential for maintaining and regenerating the synovium (11, 18). Nonetheless, while FLSs are activated and proliferate during chronic inflammatory states such as RA, they also acquire an aggressive phenotype with strong invasive properties and release ECM-degrading enzymes, thereby causing joint destruction (4, 7, 10, 17). In this study, we compared the FLS phenotypes activated during chronic inflammation in the synovium of patients with RA and those with injuries. In the LL, the percentage of pPDGFRαβ+CDH11– cells did not differ between the groups. In the SL, the percentage of pPDGFRαβ+CDH11– cells was higher in the RA group. Moreover, pPDGFRαβ+CDH11– cells exhibited resistance to cell death. Based on these observations, we hypothesized that the pPDGFRαβ+CDH11– cells in the RA-SL contributed to treatment resistance in patients with RA. Therefore, we examined the phenotypes of pPDGFRαβ+CDH11– cells in detail and identified the methods to control them.

Previous studies have shown that FLSs in the LL of RA synovial tissue exhibit pannus formation, produce MMPs and other substances, and have a direct effect on joint destruction. SL FLSs have been indicated as a possible reservoir important for supplying FLSs to the LL (7, 29). LL FLSs exhibit expression of cell-adhesion factors such as CDH11 and gp38, whereas SL FLSs express different markers such as CD90 and CD248 (7). Cells that express gp38 and CD248 have been reported to be highly sensitive to TNF-α (7, 8). The different cellular functions of FLSs in the LL and SL indicate that different therapies could be effective against FLSs. In this study, CDH11 and pPDGFRαβ expression was observed in both the LL and SL of RA synovium and acutely inflamed synovium, although we found that a larger number of pPDGFRαβ+CDH11– cells was specific to the RA-SL. In general, PDGFR+ cells in the synovium regulate synovial growth and invasion, anchorage-independent growth, and collagenase transcription in response to PDGF stimulation (17, 30). Moreover, the activation of PDGF signaling can promote cell survival and resistance to apoptosis via the PI3K–Akt pathway (14). pPDGFRαβ is activated when PDGF stimulates PDGFR (17), suggesting that the pPDGFRαβ+ cells identified in this study participated in synovial growth and invasion and accumulated in the synovium. In fact, the pPDGFRαβ+CDH11– cells that were observed in large numbers in the RA-SL were TNF-R1-negative, p16-negative, and Bcl-2-positive. TNF-R1 possesses a death domain and is characterized by an ability to induce apoptosis by activating caspase (26). In fact, the RA-SL exhibited reduced TNF-R1 expression (31), which is thought to inhibit cell death. Interestingly, the TNF-R1-positive rate in SL synovial cells was correlated negatively with the duration of RA in patients (Figure 3B). That is, prolonged affliction with RA was associated with reduced TNF-R1, which reduces the sensitivity to TNF-α. Thus, the synovium of patients with long-term RA would exhibit reduced TNF-R1 expression to help maintain chronic inflammation and promote fibrosis, which would inhibit cell death and cause cells to accumulate. p16 is an important factor in cell senescence that inhibits cell proliferation by suppressing cell cycle progression via the p16–Rb pathway (25). Reduced p16 is observed in breast cancer and other CAFs, and cells with reduced p16 expression are known to hyperproliferate and accumulate in tissues. (27) p53 suppresses cell cycle progression and promotes cell death via the p53–p21 pathway (25). Increased p53 expression in the synovium of RA causes mutations that are reported to suppress normal p53 functions in a dominant-negative manner (32, 33). As reported in this and other studies, while p53 expression is elevated, cell proliferation is not suppressed, which leads to the accumulation of cells in the synovium, suggesting that elevated p53 in the RA synovium does not function normally. Bcl-2 is involved in controlling apoptosis in the mitochondria, and Bcl-2 expression in FLSs has been correlated with synovial hyperplasia and inflammation (34). Bcl-2 expression has been observed in RA-FLSs and is thought to be associated with apoptosis resistance (26). The above findings indicate that pPDGFRαβ+CDH11– cells are resistant to cell death and are characterized by a tendency to accumulate in the synovium, suggesting that they are resistant to treatment. In contrast, pPDGFRαβ+CDH11+ cells, or cells that despite being pPDGFRαβ+ also express CDH11, exhibited an interesting feature of increased TNF-R1 and p16 expression.

CDH11 expression was observed in FLSs in healthy synovium; in addition to being involved in maintaining the shape of the synovium, CDH11 was involved in inflammation, which leads to an increase in its expression (5). CDH11 is considered an adhesion factor involved in calcium-dependent cell–cell adhesion (35), and elevated CDH11 expression in tumor cells can suppress tumor formation by inducing tumor cell apoptosis, suppressing EMT, and reducing stemness (36). Further, suppressing CDH11 expression can exacerbate the proliferation and invasion of head and neck cancer cells (37). Thus, CDH11 in tumor cells plays important roles in suppressing proliferation and invasion. Conversely, the expression of CDH11 has been reported to activate tumor cells such as breast cancer (38). In FLSs from patients with RA, CDH11 has been shown to promote the production of IL-6 and secretion of proinflammatory factors via the MAPK and NFκB signaling (39). Many of the functions of CDH 11 have not yet been elucidated, and further investigation is required in this regard. The phenotypes of aggressive RA-FLSs can have tumor-like attributes in terms of excessive proliferation, invasion, and migration capacities (10, 20), suggesting that suppressing pPDGFRαβ expression and promoting CDH11 expression in FLSs could be an effective treatment strategy for RA. Further, reducing the number of FLSs that accumulate specifically in the RA-SL is necessary.

We conducted an *in vitro* experiment to identify effective therapies related to pPDGFRαβ and CDH11 expression in FLSs. Rosengren et al. (20) increased the synergistic response of FLSs by including 2GF stimulation *in vitro* and showed that the addition of TNF-α increased the production of IL-6, IL-8, and MMP3. Similarly, Shibuya et al. (21) stimulated FLSs with 2GF + TNF, which increased PI3Kδ expression and led to synovial hyperplasia by activating the PI3K–Akt pathway. In other words, the phenotype obtained by stimulating FLSs with 2GF + TNF resembled that of the pPDGFRαβ+CDH11– cells that were observed *in vivo*. Based on the pPDGFRαβ/CDH11 ratios, we found that stimulating FLSs obtained from RA with 2GF caused the ratio to decrease, whereas stimulation with 2GF + TNF caused it to increase. PDGF-BB, TGF-β, and TNF-α are abundant in the synovial environment of patients with RA (40, 41). Increased expression of PDGF-B and TGF-β has been reported during acute inflammation of the synovium, such as due to injury (42). Further, TNF-α expression is known to be stronger in RA synovium compared to that in the synovium during acute inflammation (41). Accordingly, *in vitro* 2GF + TNF stimulation is thought to be a reliable model that accurately reflects the synovial environment of RA and is useful for assessing the therapeutic effects against pPDGFRαβ+CDH11– cells. We found that a TNF inhibitor reduced the pPDGFRαβ/CDH11 ratio, which was elevated by 2GF + TNF stimulation. We believe that the decrease in the pPDGFRαβ/CDH11 ratio occurred because the TNF inhibitor suppressed TNF signaling and interactions between PDGFR and CDH11 occurred. In a previous study, TNF-α stimulation was shown to increase PDGFR expression via MAPK and c-Src (43). Moreover, although the detailed mechanism remains unclear, TNF-α stimulation has been reported to create a feedback loop in which increased Rsk2 expression suppresses TNF signaling and CDH11 expression (44). In fact, TNF inhibitors have been shown to have high efficacy against RA in humans by suppressing TNF-α and inducing cell death in FLSs (45). SL fibrosis has been reported to occur when TNF inhibitors are used in RA therapy (12). Although TNF inhibitor therapy can be expected to prevent cell accumulation, it might only marginally affect pre-existing cell accumulation (46).

We believe that reducing the accumulated FLSs is essential for successful RA therapy. In this study, excessive cell accumulation was noted in the SL, and many of these cells were pPDGFRαβ+CDH11–. Moreover, the percentage of p16-positive pPDGFRαβ+CDH11– cells was low. The p16 protein inhibits cell proliferation by suppressing cell cycle progression and negatively controlling CDK4/6 (25). Therefore, we surmised that suppressing CDK4/6 would inhibit the proliferation of pPDGFRαβ+CDH11– cells. A large-scale genome-wide association study showed that CDK4/6 was a risk gene for RA, raising the possibility that CDK4/6 inhibitors could be effective therapeutics for RA (47). Moreover, the CDK4/6 inhibitor palbociclib suppressed arthritis in RA animal models (23, 48). Therefore, we hypothesized that palbociclib could potentially suppress FLS proliferation and reduce the cell count, both of which were confirmed experimentally. However, palbociclib was not found to reduce the pPDGFRαβ/CDH11 ratio. Next, we tested combination treatment with a TNF inhibitor and CDK4/6 inhibitor, which decreased the cell count, but did not decrease the pPDGFRαβ/CDH11 ratio. Administering CDK4/6 for treating breast cancer has been reported to increase TNF-α production and antigen presentation (49). In this study, the effects of CDK4/6 might have amplified the action of TNF-α to mask the effects of the TNF inhibitor.

The interesting finding of this study was the cell population that characteristically showed increased pPDGFRαβ+CDH11– expression in the RA SL. pPDGFRαβ+CDH11– cells exhibit cell death resistance and abnormal proliferation, which suggests a tendency to accumulate in the synovium. Other reported FLS markers include CD90, CD248, and gp38 (7, 8). We were unable to determine whether the pPDGFRαβ+CDH11– cells identified in this study differed from cell populations identified with other makers. However, we believe that pPDGFRαβ+CDH11– cells were almost mesenchymal cells because, among the total 8.3% pPDGFRαβ+CDH11– cells, 1.3% were CD45+ hematopoietic cells, the percentage of which was very small (Supplemental Figure 2). Furthermore, cells that express gp38 and CD248 have been reported to be highly sensitive to TNF-α (8, 50), indicating that they were different cell populations than pPDGFRαβ+CDH11– cells, which have low TNF-R1 expression. Moreover, CD90 is found not only in RA-FLSs, but is a cell-surface marker that is also widely expressed in normal FLSs (19), suggesting a high likelihood of expression in pPDGFRαβ+CDH11– cells. The pPDGFRαβ used in this study was activated by PDGF signaling. Previous studies have shown that suppressing PDGFR suppresses FLSs (17), indicating that pPDGFRαβ is a marker that is intimately related to FLS functions. Further, the control samples used in this study were obtained from post-traumatic synovium, such as after fracture or an ACL injury. We used post-traumatic synovium because our objective was to compare synovium in the acute and chronic inflammatory states to clarify the characteristics of the cells that accumulated. With post-traumatic synovium, the control group was younger and contained more men than in the RA group, indicating that the groups were not matched for age or sex. Nevertheless, the percentages of cells expressing pPDGFRαβ and/or CDH11 in this study did not correlate with age or sex (Figures 1E–G, Supplemental Figure 6). Thus, although we cannot rule out the influence of age or sex, the increase in pPDGFRαβ+CDH11– cells in the RA-SL might not have been affected by age or sex and was highly likely a cause of pathology in RA. In this study, we also found that TNF inhibitors increased the expression level of CDH11. Such an increase appeared to decrease Bcl-2 expression and increase p16 expression, thereby suppressing the resistance to cell death and causing excessive cell proliferation of RA-FLSs. Although TNF inhibitors exert a strong therapeutic effect clinically, treatment-resistant cases are common (3). In particular, while TNF inhibitors are reported to act on the LL, they tend to have less effect on the SL (9). Because the cells targeted in this study are abundant in the SL, enabling the pharmacological effects of TNF inhibitors to reach the SL could be important for maximizing their therapeutic effects. Future *in vivo* experiments are warranted to identify effective therapies that target pPDGFRαβ+CDH11– cells accumulating in the SL, including the examination of drug delivery to the SL.

In this study, we showed that pPDGFRαβ+CDH11– cells accumulated specifically in the SL of the synovium in RA, and that their resistance to cell death could lead to the accumulation of synovial cells and resistance to treatment. Stimulation with 2GF + TNF caused the cells to remain in a pPDGFRαβ-predominant state, similar to the pPDGFRαβ+CDH11– cells observed in RA-SL, which indicates that therapies that are effective against these cells could lead to treatments for cells accumulated in the synovium. Clarifying the characteristics of treating FLSs with TNF or CDK4/6 inhibitors and using drugs in ways that are adapted to the pathology of the synovium could lead to better therapeutic effects.

## Author Contributions

TM, YS, and TS-C designed the experiments, performed the experiments, analyzed the data, and drafted the manuscript. TS, AT, and YO obtained tissue samples from patients. TY and MF coauthored the paper.

### Conflict of Interest Statement

The authors declare that the research was conducted in the absence of any commercial or financial relationships that could be construed as a potential conflict of interest.

## Supplementary Material

Supplemental Figure 1 The quantitative data show the cell density of pPDGFRαβ+CDH11−, pPDGFRαβ−CDH11+, and pPDGFRαβ+CDH11+ cells. The cell densities of pPDGFRαβ+ CDH11−, pPDGFRαβ−CDH11+, and pPDGFRαβ+CDH11+ cells in the LL were not different between RA and control groups. However, their densities in the SL were significantly higher in the RA group. An unpaired *t*-test was used for statistical analysis. The significance level was *P* < 0.05.

Supplemental Figure 2 The percentages of cells expressing pPDGFRαβ, CDH11, and CD45. Representative images of pPDGFRαβ, CDH11, and CD45 staining and the percentages are shown in the pie chart.

Supplemental Figure 3 Comparison between the RA and the control group for quantitative expression data for Bcl-2 and p16 in 3 cell populations (pPDGFRαβ+CDH11−, pPDGFRαβ−CDH11+, pPDGFRαβ+CDH11+ cells) in the SL of the synovium. One-way ANOVA was used for statistical analysis. The significance level was *P* < 0.05.

Supplemental Figure 4 Cell proliferation assay by using PDGF-BB, TGF-β, and TNF-α stimulation of RA-FLS. The stimulation with PDGF-BB, TGF-β, and TNF-α stimulation did not show significant difference in RA-FLS. One-way ANOVA was used for statistical analysis. The significance level was *P* < 0.05.

Supplemental Figure 5 Normalized expression of pPDGFRαβ and CDH11 expression by using 2GF + TNF, and etanercept and palbociclib in RA-FLSs. **(A,B)** Normalized expression of pPDGFRαβ and CDH11 in RA-FLSs stimulated with PDGF-BB, TGF-β, and TNF-α in each combination. **(C,D)** Normalized expression of pPDGFRαβ and CDH11 in RA-FLSs stimulated with 2GF + TNF, and etanercept and palbociclib in each combination. One-way ANOVA was used for statistical analysis. The significance level was *P* < 0.05.

Supplemental Figure 6 Correlation between the percentages of cells expressing pPDGFRαβ and/or CDH11, and the characteristics of patients. The percentages of cells expressing pPDGFRαβ and/or CDH11 did not correlate with age or sex. Correlations were examined statistically by using Pearson's correlation coefficient. The significance level was *P* < 0.05.

## Abbreviations

RA: rheumatoid arthritis
FLSs: fibroblast-like synoviocytes
CDH11: cadherin-11
LL: lining layer
SL: sub-lining layer
MMPs: matrix metalloproteinases
TNF: tumor necrosis factor
PDGF: platelet-derived growth factor
Bcl-2: B-cell lymphoma 2
HE: hematoxylin and eosin
PBS: phosphate-buffered saline
BSA: bovine serum albumin
RT: room temperature
ICCs: intraclass correlation coefficients
PSL: prednisolone
MTX: methotrexate
CAFs: cancer-associated fibroblasts
EMT: epithelial-mesenchymal transition
ECM: extracellular matrix.

## References

[1] McInnes IB Schett G. The pathogenesis of rheumatoid arthritis. N Engl J Med. (2011) 365:2205–19. 10.1056/NEJMra1004965 22150039

[2] Brown AK Conaghan PG Karim Z Quinn MA Ikeda K Peterfy CG. An explanation for the apparent dissociation between clinical remission and continued structural deterioration in rheumatoid arthritis. Arthritis Rheum. (2008) 58:2958–67. 10.1002/art.23945 18821687

[3] Yoshida K Kishimoto M Radner H Matsui K Okada M Saeki Y. Low rates of biologic-free clinical disease activity index remission maintenance after biologic disease-modifying anti-rheumatic drug discontinuation while in remission in a Japanese multicentre rheumatoid arthritis registry. Rheumatology (2016) 55:286–90. 10.1093/rheumatology/kev329 26350484 PMC4751227

[4] Bottini N Firestein GS. Duality of fibroblast-like synoviocytes in RA: passive responders and imprinted aggressors. Nat Rev Rheumatol. (2013) 9:24–33. 10.1038/nrrheum.2012.190 23147896 PMC3970924

[5] Lee DM Kiener HP Agarwal SK Noss EH Watts GF Chisaka O. Cadherin-11 in synovial lining formation and pathology in arthritis. Science (2007) 315:1006–10. 10.1126/science.1137306 17255475

[6] Iwanaga T Shikichi M Kitamura H Yanase H Nozawa-Inoue K. Morphology and functional roles of synoviocytes in the joint. Arch Histol Cytol. (2000) 63:17–31. 10.1679/aohc.63.17 10770586

[7] Ospelt C. Synovial fibroblasts in 2017. RMD Open (2017) 3:e000471. 10.1136/rmdopen-2017-000471 29081987 PMC5652455

[8] Croft AP Naylor AJ Marshall JL Hardie DL Zimmermann B Turner J. Rheumatoid synovial fibroblasts differentiate into distinct subsets in the presence of cytokines and cartilage. Arthritis Res Ther. (2016) 18:270. 10.1186/s13075-016-1156-1 27863512 PMC5116193

[9] Vandooren B Cantaert T ter Borg M Noordenbos T Kuhlman R Gerlag D. Tumor necrosis factor alpha drives cadherin 11 expression in rheumatoid inflammation. Arthritis Rheum. (2008) 58:3051–62. 10.1002/art.23886 18821672

[10] Bartok B Firestein GS. Fibroblast-like synoviocytes: key effector cells in rheumatoid arthritis. Immunol Rev. (2010) 233:233–55. 10.1111/j.0105-2896.2009.00859.x 20193003 PMC2913689

[11] Buckley CD Pilling D Lord JM Akbar AN Scheel-Toellner D Salmon M. Fibroblasts regulate the switch from acute resolving to chronic persistent inflammation. Trends Immunol. (2001) 22:199–204. 10.1016/S1471-4906(01)01863-4 11274925

[12] Hirohata S Tomita T Yoshikawa H Kyogoku M. TNF inhibitors induce discoid fibrosis in the sublining layers of the synovium with degeneration of synoviocytes in rheumatoid arthritis. Rheumatol Int. (2013) 33:2473–81. 10.1007/s00296-013-2743-y 23575549 PMC3782653

[13] Izquierdo E Canete JD Celis R Del Rey MJ Usategui A Marsal S. Synovial fibroblast hyperplasia in rheumatoid arthritis: clinicopathologic correlations and partial reversal by anti-tumor necrosis factor therapy. Arthritis Rheum. (2011) 63:2575–83. 10.1002/art.30433 21547893

[14] Ying HZ Chen Q Zhang WY Zhang HH Ma Y Zhang SZ. PDGF signaling pathway in hepatic fibrosis pathogenesis and therapeutics (Review). Mol Med Rep. (2017) 16:7879–89. 10.3892/mmr.2017.7641 28983598 PMC5779870

[15] Uezumi A Fukada S Yamamoto N Ikemoto-Uezumi M Nakatani M Morita M. Identification and characterization of PDGFRalpha^+^ mesenchymal progenitors in human skeletal muscle. Cell Death Dis. (2014) 5:e1186. 10.1038/cddis.2014.161 24743741 PMC4001314

[16] Saito Y Chikenji T Ozasa Y Fujimiya M Yamashita T Gingery A. PDGFR signaling mediates hyperproliferation and fibrotic responses of subsynovial connective tissue cells in idiopathic carpal tunnel syndrome. Sci Rep. (2017) 7:16192. 10.1038/s41598-017-16443-w 29170419 PMC5700922

[17] Charbonneau M Lavoie RR Lauzier A Harper K McDonald PP Dubois CM. Platelet-derived growth factor receptor activation promotes the prodestructive invadosome-forming phenotype of synoviocytes from patients with rheumatoid arthritis. J Immunol. (2016) 196:3264–75. 10.4049/jimmunol.1500502 26976956

[18] Lieberthal J Sambamurthy N Scanzello CR. Inflammation in joint injury and post-traumatic osteoarthritis. Osteoarthritis Cartilage (2015) 23:1825–34. 10.1016/j.joca.2015.08.015 26521728 PMC4630675

[19] Rosengren S Boyle DL Firestein GS. Acquisition, culture, and phenotyping of synovial fibroblasts. In: Cope AP, editor. Methods in Molecular Medicine, Vol. 135. Totowa, NJ: Humana Press Inc. (2007). p. 365–75. 10.1007/978-1-59745-401-8_24 17951672

[20] Rosengren S Corr M Boyle DL. Platelet-derived growth factor and transforming growth factor beta synergistically potentiate inflammatory mediator synthesis by fibroblast-like synoviocytes. Arthritis Res Ther. (2010) 12:R65. 10.1186/ar2981 20380722 PMC2888219

[21] Shibuya H Yoshitomi H Murata K Kobayashi S Furu M Ishikawa M. TNFα, PDGF, and TGFβ synergistically induce synovial lining hyperplasia via inducible PI3Kδ. Mod Rheumatol. (2015) 25:72–8. 10.3109/14397595.2014.900847 24716596

[22] Qin G Xu F Qin T Zheng Q Shi D Xia W. Palbociclib inhibits epithelial-mesenchymal transition and metastasis in breast cancer via c-Jun/COX-2 signaling pathway. Oncotarget (2015) 6:41794–808. 10.18632/oncotarget.5993 26540629 PMC4747189

[23] Sekine C Sugihara T Miyake S Hirai H Yoshida M Miyasaka N. Successful treatment of animal models of rheumatoid arthritis with small-molecule cyclin-dependent kinase inhibitors. J Immunol. (2008) 180:1954–61. 10.4049/jimmunol.180.3.1954 18209094

[24] Kanda Y. Investigation of the freely available easy-to-use software 'EZR' for medical statistics. Bone Marrow Transplant. (2013) 48:452–8. 10.1038/bmt.2012.244 23208313 PMC3590441

[25] Childs BG Baker DJ Kirkland JL Campisi J van Deursen JM. Senescence and apoptosis: dueling or complementary cell fates? EMBO Rep. (2014) 15:1139–53. 10.15252/embr.201439245 25312810 PMC4253488

[26] Korb A Pavenstadt H Pap T. Cell death in rheumatoid arthritis. Apoptosis (2009) 14:447–54. 10.1007/s10495-009-0317-y 19199037

[27] Al-Ansari MM Hendrayani SF Shehata AI Aboussekhra A. p16(INK4A) represses the paracrine tumor-promoting effects of breast stromal fibroblasts. Oncogene (2013) 32:2356–64. 10.1038/onc.2012.270 22751126 PMC3679618

[28] Reed AL Califano J Cairns P Westra WH Jones RM Koch W. High frequency of p16 (CDKN2/MTS-1/INK4A) inactivation in head and neck squamous cell carcinoma. Cancer Res. (1996) 56:3630–3. 8705996

[29] Kiener HP Karonitsch T. The synovium as a privileged site in rheumatoid arthritis: cadherin-11 as a dominant player in synovial pathology. Best Pract Res Clin Rheumatol. (2011) 25:767–77. 10.1016/j.berh.2011.11.012 22265259

[30] Lafyatis R Remmers EF Roberts AB Yocum DE Sporn MB Wilder RL Anchorage-independent growth of synoviocytes from arthritic and normal joints. Stimulation by exogenous platelet-derived growth factor and inhibition by transforming growth factor-beta and retinoids. J Clin Invest (1989) 83:1267-76. 10.1172/JCI114011 PMC303817 2784799

[31] Alsalameh S Winter K Al-Ward R Wendler J Kalden JR Kinne RW. Distribution of TNF-alpha, TNF-R55 and TNF-R75 in the rheumatoid synovial membrane: TNF receptors are localized preferentially in the lining layer; TNF-alpha is distributed mainly in the vicinity of TNF receptors in the deeper layers. Scand J Immunol. (1999) 49:278–85. 10.1046/j.1365-3083.1999.00458.x 10102645

[32] Han Z Boyle DL Shi Y Green DR Firestein GS. Dominant-negative p53 mutations in rheumatoid arthritis. Arthritis Rheum. (1999) 42:1088–92. 10.1002/1529-0131(199906)42:6<1088::AID-ANR4>3.0.CO;2-E 10366100

[33] Tak PP Smeets TJ Boyle DL Kraan MC Shi Y Zhuang S. p53 overexpression in synovial tissue from patients with early and longstanding rheumatoid arthritis compared with patients with reactive arthritis and osteoarthritis. Arthritis Rheum. (1999) 42:948–53. 10.1002/1529-0131(199905)42:5<948::AID-ANR13>3.0.CO;2-L 10323450

[34] Perlman H Georganas C Pagliari LJ Koch AE Haines K Pope RM. Bcl-2 expression in synovial fibroblasts is essential for maintaining mitochondrial homeostasis and cell viability. J Immunol. (2000) 164:5227–35. 10.4049/jimmunol.164.10.5227 10799883

[35] Feltes CM Kudo A Blaschuk O Byers SW. An alternatively spliced cadherin-11 enhances human breast cancer cell invasion. Cancer Res. (2002) 62:6688–97. Available online at: http://cancerres.aacrjournals.org/content/62/22/6688 12438268

[36] Li L Ying J Li H Zhang Y Shu X Fan Y. The human cadherin 11 is a pro-apoptotic tumor suppressor modulating cell stemness through Wnt/beta-catenin signaling and silenced in common carcinomas. Oncogene (2012) 31:3901–12. 10.1038/onc.2011.541 22139084 PMC3426851

[37] Piao S Inglehart RC Scanlon CS Russo N Banerjee R D'Silva NJ. CDH11 inhibits proliferation and invasion in head and neck cancer. J Oral Pathol Med. (2017) 46:89–97. 10.1111/jop.12471 27397103

[38] Pishvaian MJ Feltes CM Thompson P Bussemakers MJ Schalken JA Byers SW. Cadherin-11 is expressed in invasive breast cancer cell lines. Cancer Res. (1999) 59:947–52. Available online at: http://cancerres.aacrjournals.org/content/59/4/947 10029089

[39] Chang SK Noss EH Chen M Gu Z Townsend K Grenha R. Cadherin-11 regulates fibroblast inflammation. Proc Natl Acad Sci USA. (2011) 108:8402–7. 10.1073/pnas.1019437108 21536877 PMC3100978

[40] Monier S Reme T Cognot C Gao QL Travaglio-Encinoza A Cuchacovich M. Growth factor activity of IL-6 in the synovial fluid of patients with rheumatoid arthritis. Clin Exp Rheumatol. (1994) 12:595–602. 7895392

[41] Santos Savio A Machado Diaz AC Chico Capote A Miranda Navarro J Rodriguez Alvarez Y Bringas Perez R Differential expression of pro-inflammatory cytokines IL-15Ralpha, IL-15, IL-6 and TNFalpha in synovial fluid from rheumatoid arthritis patients. BMC Musculoskelet Disord. (2015) 16:51 10.1186/s12891-015-0516-3 25879761 PMC4359511

[42] Hayward AL Deehan DJ Aspden RM Sutherland AG. Analysis of sequential cytokine release after ACL reconstruction. Knee Surg Sports Traumatol Arthrosc. (2011) 19:1709–15. 10.1007/s00167-011-1486-0 21445592

[43] Tsai CL Chen WC Lee IT Chi PL Cheng SE Yang CM. c-Src-dependent transactivation of PDGFR contributes to TNF-alpha-induced MMP-9 expression and functional impairment in osteoblasts. Bone (2014) 60:186–97. 10.1016/j.bone.2013.12.014 24361597

[44] Derer A Bohm C Grotsch B Grun JR Grutzkau A Stock M. Rsk2 controls synovial fibroblast hyperplasia and the course of arthritis. Ann Rheum Dis. (2016) 75:413–21. 10.1136/annrheumdis-2014-205618 25414238

[45] Pattacini L Boiardi L Casali B Salvarani C. Differential effects of anti-TNF-alpha drugs on fibroblast-like synoviocyte apoptosis. Rheumatology (2010) 49:480–9. 10.1093/rheumatology/kep358 20040530

[46] Scheel T Gursche A Zacher J Haupl T Berek C. V-region gene analysis of locally defined synovial B and plasma cells reveals selected B cell expansion and accumulation of plasma cell clones in rheumatoid arthritis. Arthritis Rheum. (2011) 63:63–72. 10.1002/art.27767 20882667

[47] Okada Y Wu D Trynka G Raj T Terao C Ikari K. Genetics of rheumatoid arthritis contributes to biology and drug discovery. Nature (2014) 506:376–81. 10.1038/nature12873 24390342 PMC3944098

[48] Hosoya T Iwai H Yamaguchi Y Kawahata K Miyasaka N Kohsaka H. Cell cycle regulation therapy combined with cytokine blockade enhances antiarthritic effects without increasing immune suppression. Ann Rheum Dis. (2016) 75:253–9. 10.1136/annrheumdis-2014-205566 25165034

[49] Goel S DeCristo MJ Watt AC BrinJones H Sceneay J Li BB. CDK4/6 inhibition triggers anti-tumour immunity. Nature (2017) 548:471–5. 10.1038/nature23465 28813415 PMC5570667

[50] Hardy RS Hulso C Liu Y Gasparini SJ Fong-Yee C Tu J. Characterisation of fibroblast-like synoviocytes from a murine model of joint inflammation. Arthritis Res Ther. (2013) 15:R24. 10.1186/ar4158 23363614 PMC3672796

