# Supplementary figures and images for: Phosphorylated Platelet-Derived Growth Factor Receptor-Positive Cells With Anti-apoptotic Properties Accumulate in the Synovium of Patients With Rheumatoid Arthritis
Source: Front Immunol. 2019 Feb 15;10:241. doi: 10.3389/fimmu.2019.00241 (PMC6384265; doi:10.3389/fimmu.2019.00241)

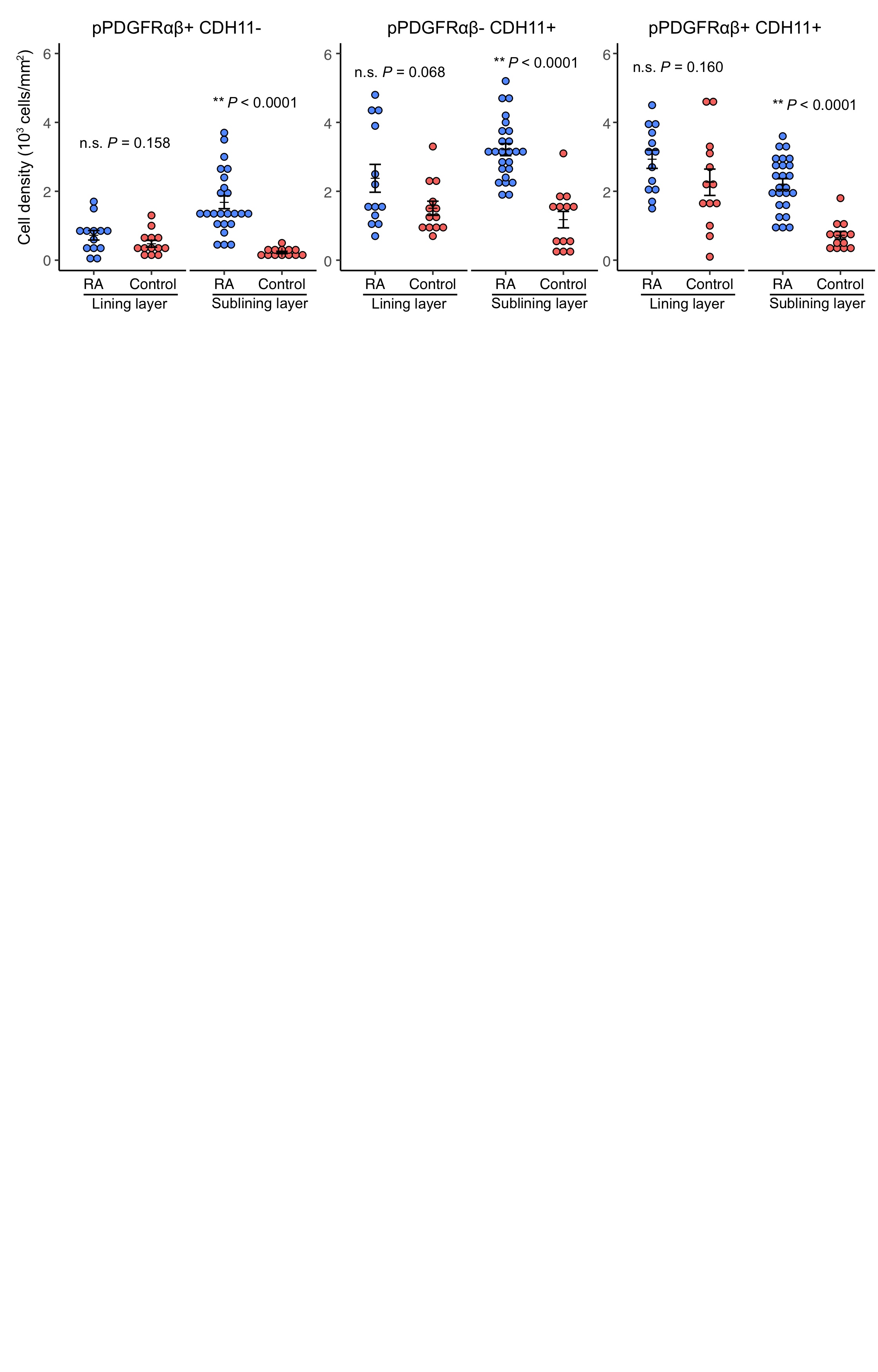

Supplement: Supplemental Figure 1 — The quantitative data show the cell density of pPDGFRαβ+CDH11−, pPDGFRαβ−CDH11+, and pPDGFRαβ+CDH11+ cells. The cell densities of pPDGFRαβ+ CDH11−, pPDGFRαβ−CDH11+, and pPDGFRαβ+CDH11+ cells in the LL were not different between RA and control groups. However, their densities in the SL were significantly higher in the RA group. An unpaired t-test was used for statistical analysis. The significance level was P < 0.05. [file Image_1.JPEG]

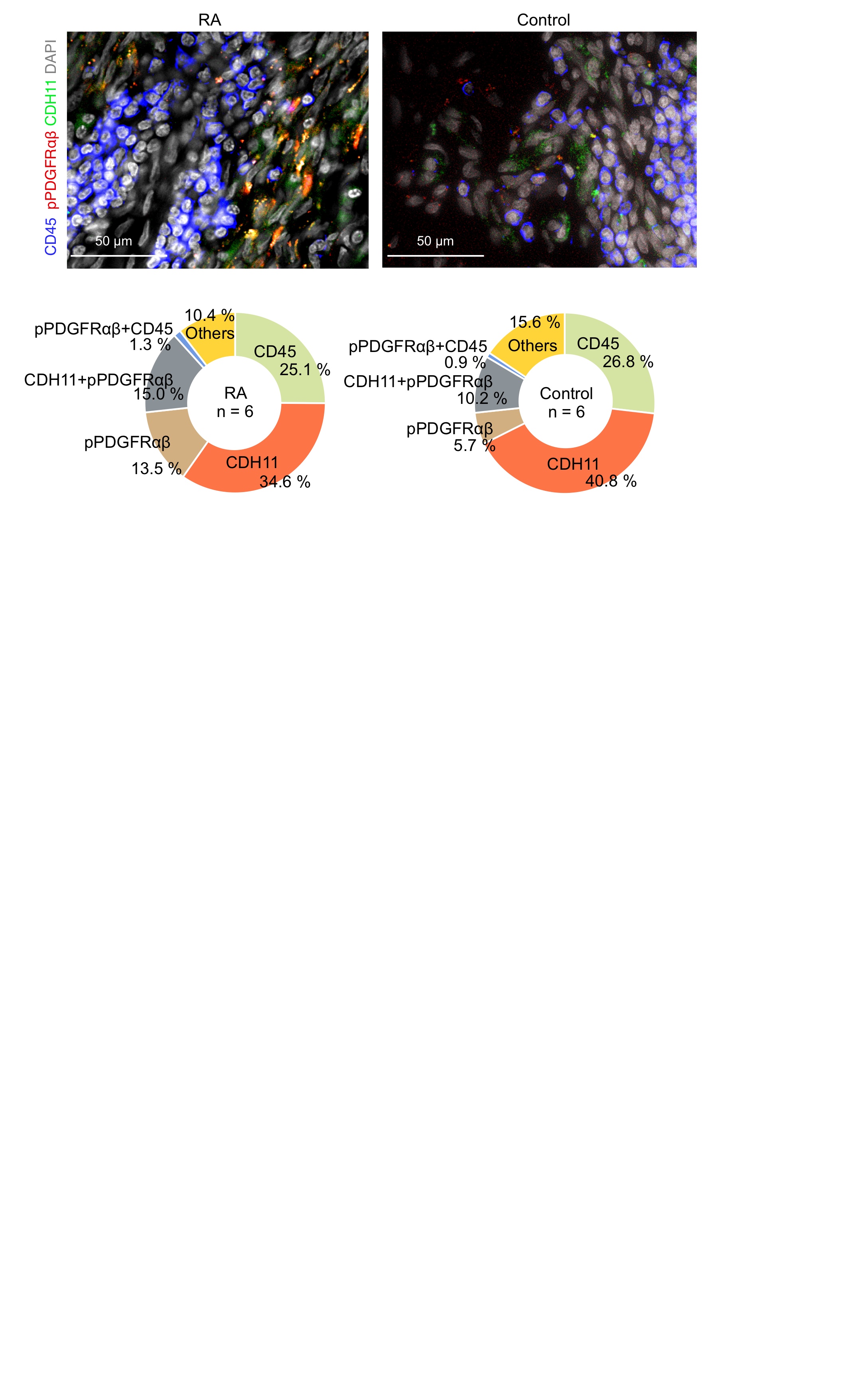

Supplement: Supplemental Figure 2 — The percentages of cells expressing pPDGFRαβ, CDH11, and CD45. Representative images of pPDGFRαβ, CDH11, and CD45 staining and the percentages are shown in the pie chart. [file Image_2.JPEG]

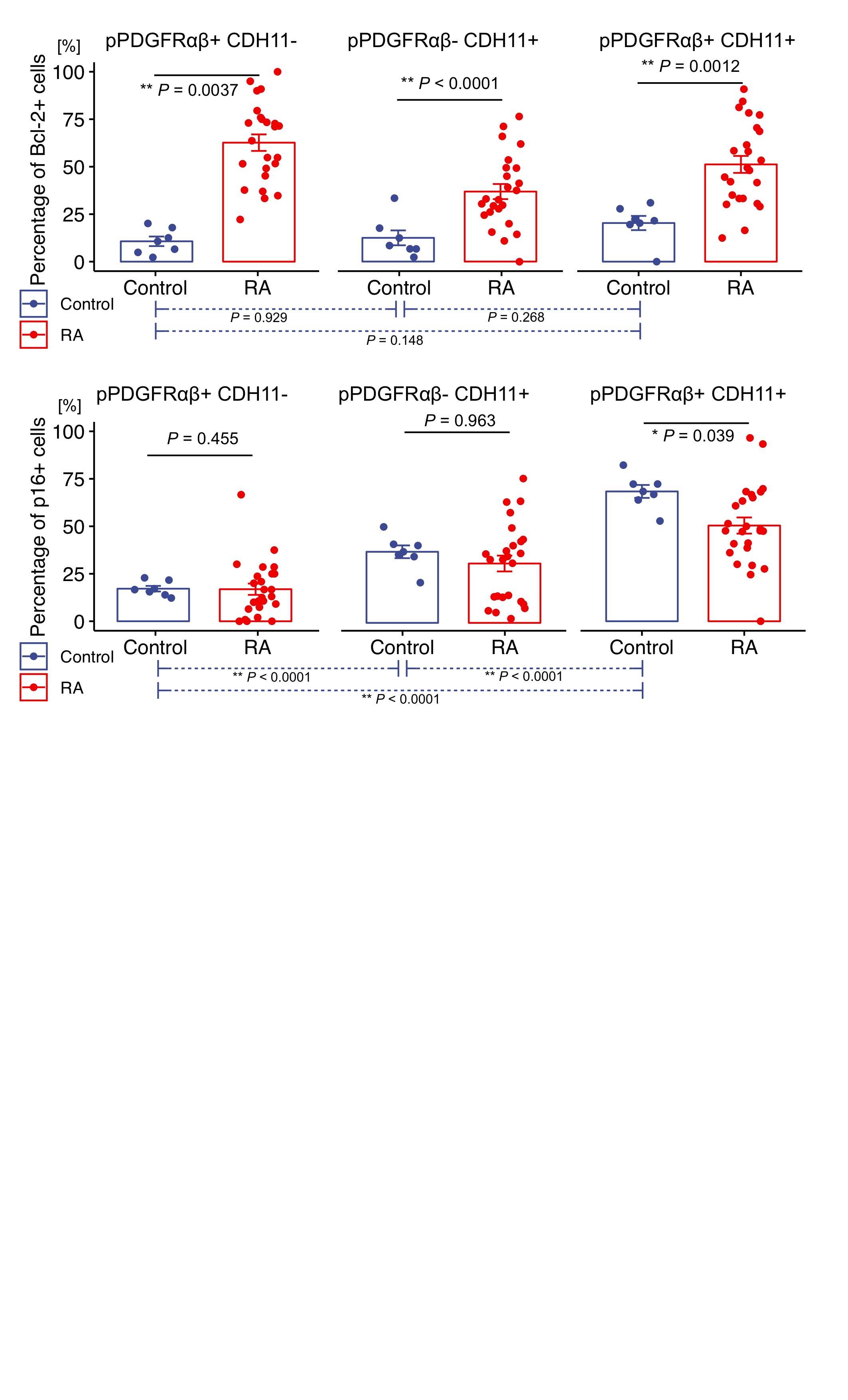

Supplement: Supplemental Figure 3 — Comparison between the RA and the control group for quantitative expression data for Bcl-2 and p16 in 3 cell populations (pPDGFRαβ+CDH11−, pPDGFRαβ−CDH11+, pPDGFRαβ+CDH11+ cells) in the SL of the synovium. One-way ANOVA was used for statistical analysis. The significance level was P < 0.05. [file Image_3.JPEG]

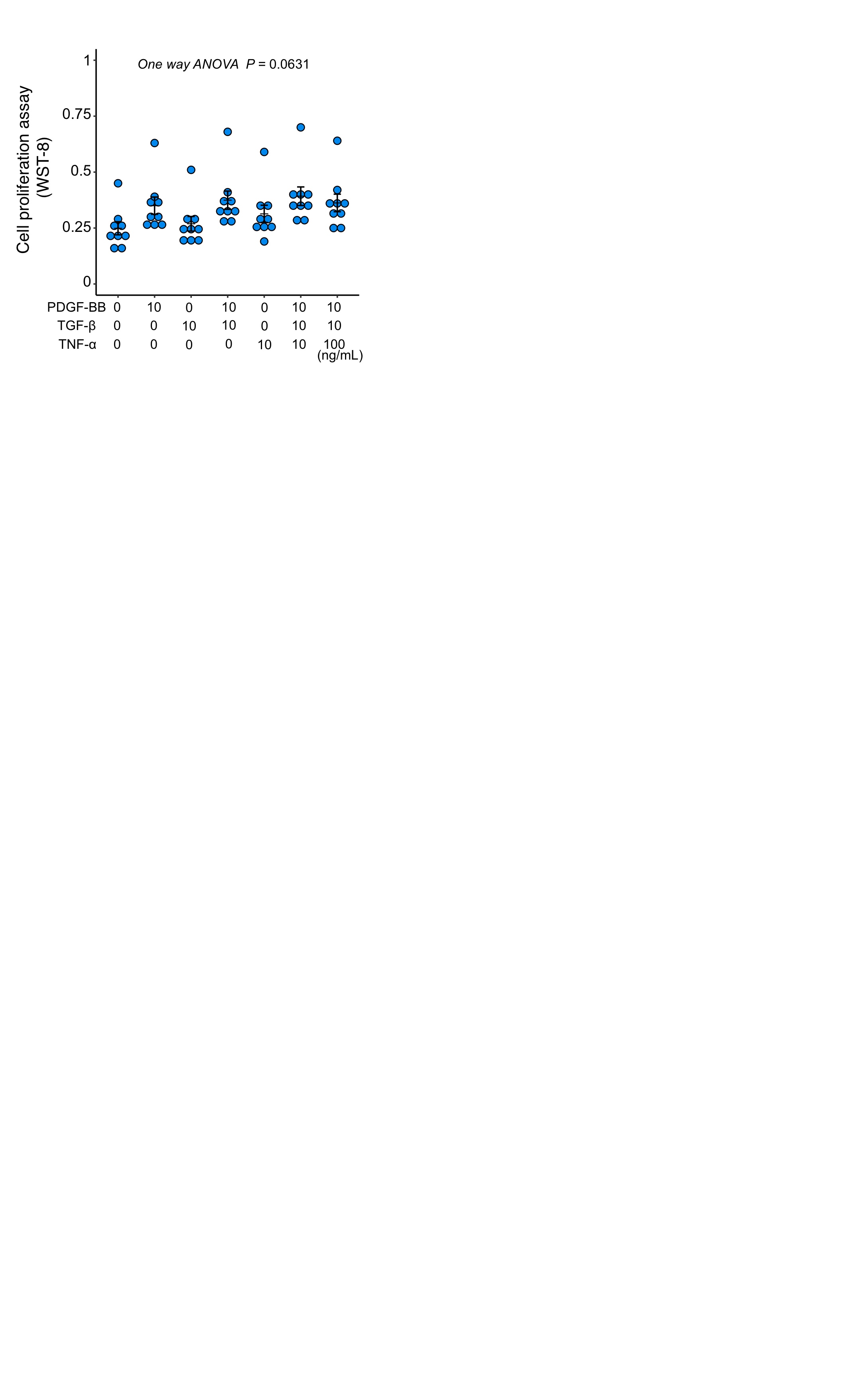

Supplement: Supplemental Figure 4 — Cell proliferation assay by using PDGF-BB, TGF-β, and TNF-α stimulation of RA-FLS. The stimulation with PDGF-BB, TGF-β, and TNF-α stimulation did not show significant difference in RA-FLS. One-way ANOVA was used for statistical analysis. The significance level was P < 0.05. [file Image_4.JPEG]

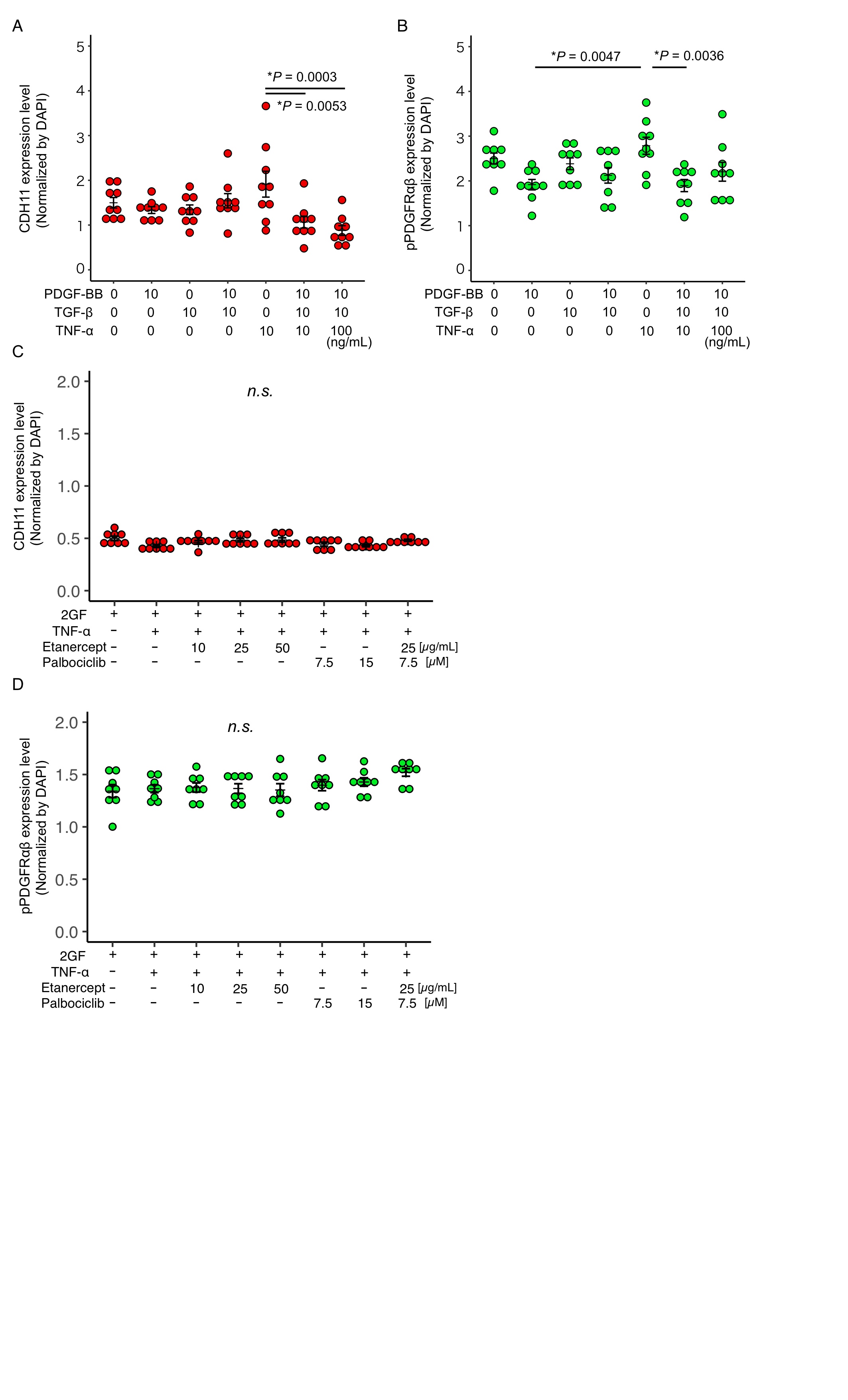

Supplement: Supplemental Figure 5 — Normalized expression of pPDGFRαβ and CDH11 expression by using 2GF + TNF, and etanercept and palbociclib in RA-FLSs. (A,B) Normalized expression of pPDGFRαβ and CDH11 in RA-FLSs stimulated with PDGF-BB, TGF-β, and TNF-α in each combination. (C,D) Normalized expression of pPDGFRαβ and CDH11 in RA-FLSs stimulated with 2GF + TNF, and etanercept and palbociclib in each combination. One-way ANOVA was used for statistical analysis. The significance level was P < 0.05. [file Image_5.JPEG]

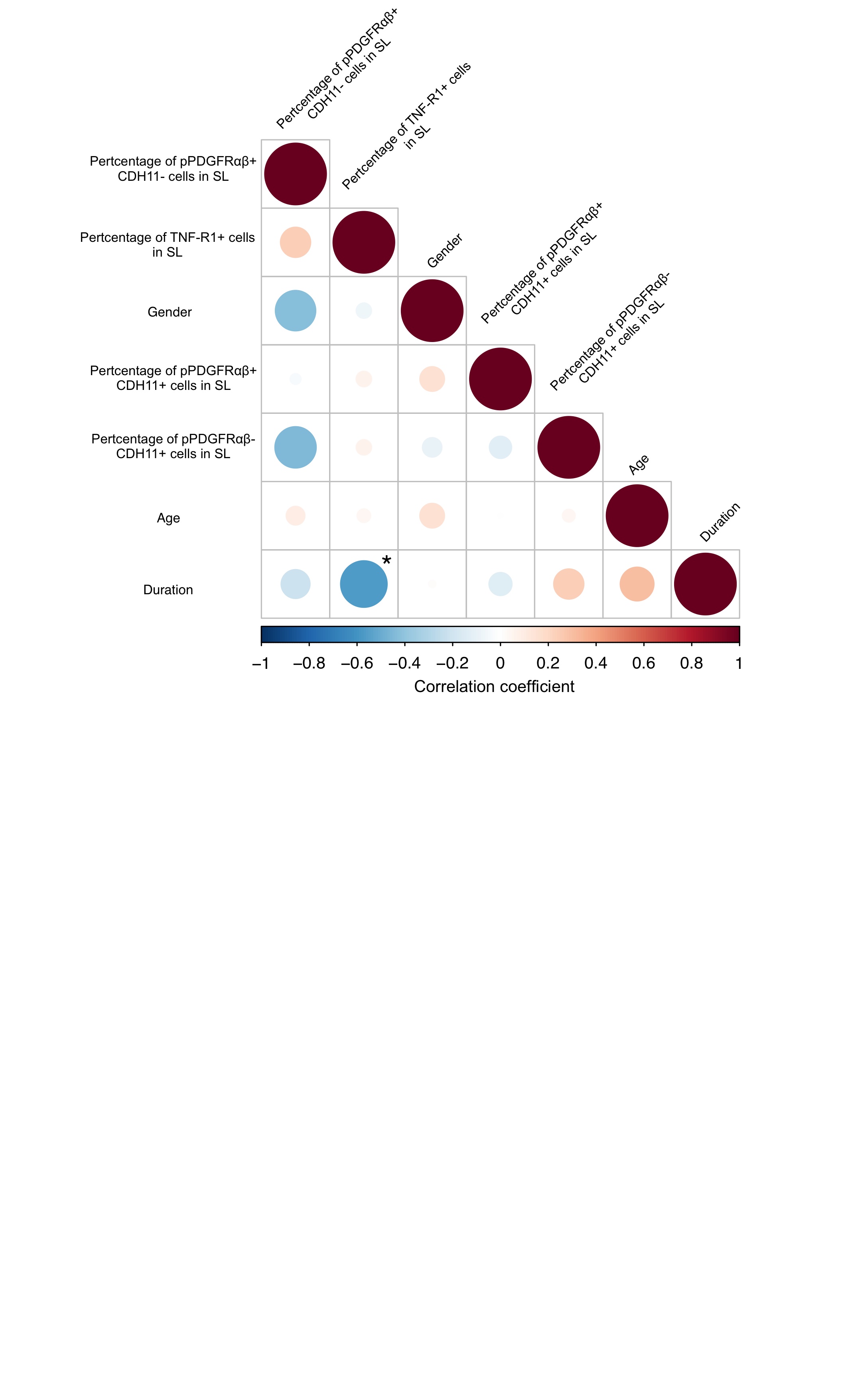

Supplement: Supplemental Figure 6 — Correlation between the percentages of cells expressing pPDGFRαβ and/or CDH11, and the characteristics of patients. The percentages of cells expressing pPDGFRαβ and/or CDH11 did not correlate with age or sex. Correlations were examined statistically by using Pearson's correlation coefficient. The significance level was P < 0.05. [file Image_6.JPEG]
